# Epidemiological factors associated with Turtle fraservirus 1 (TFV1) in freshwater turtles in Florida, USA

**DOI:** 10.1371/journal.pone.0320097

**Published:** 2025-04-01

**Authors:** Lisa A. Shender, Andrea Sylvia, Brian A. Stacy, Veronica Guzman-Vargas, Thais C.S. Rodrigues, Pedro Viadanna, Jordan A. Vann, Kylle Cahill-Patray, Thomas B. Waltzek, Kuttichantran Subramanium, Brittany Bankovich

**Affiliations:** 1 Wildlife Health, Fish and Wildlife Research Institute, Florida Fish and Wildlife Conservation Commission, Gainesville, Florida, United States of America; 2 Center for Biostatistics and Modeling, Fish and Wildlife Research Institute, Florida Fish and Wildlife Conservation Commission, Gainesville, Florida, United States of America; 3 Office of Protected Resources, National Oceanic and Atmospheric Administration Fisheries, Gainesville, Florida, United States of America; 4 Department of Infectious Diseases and Immunology, College of Veterinary Medicine, University of Florida, Gainesville, Florida, United States of America; 5 Center for Spatial Analysis, Fish and Wildlife Research Institute, Florida Fish and Wildlife Conservation Commission, Gainesville, Florida, United States of America; MARE – Marine and Environmental Sciences Centre, PORTUGAL

## Abstract

Turtle fraservirus 1 (TFV1) is an emerging pathogen that was first discovered in freshwater turtles in peninsular Florida (USA) in 2018. The incubation period, transmission route(s), range of virus-susceptible species, and other key epidemiological factors that pertain to this disease are still unknown. Therefore, the primary aims of this work were to 1) evaluate TFV1 infection and available metadata using an epidemiological framework and 2) summarize our findings into Florida-specific guidance for turtle morbidity (e.g., diseased condition) and mortality investigations by managers faced with limited resources. This study included several species of sick or dead freshwater turtles collected from 9 March 2018 until 5 September 2021. These collections were greatly facilitated by public reporting and submissions from state-permitted wildlife rehabilitation centers. To evaluate data obtained from different stages of a mortality investigation, we developed four datasets pertaining to field collection, necropsy findings, weather conditions, and spatial and temporal patterns. Within each dataset, we used logistic regression to determine the relative effect of available explanatory variables on the probability of a TFV1-positive PCR test result. We found that >50% (47/93) turtles tested positive for TFV1. The presence of cloacal and/or oral plaques in softshell turtles was strongly associated with TFV1-positive infection status. Furthermore, turtles that were collected from clustered mortality events (>1 turtle found sick or deceased) were more likely to test positive, with both distance and time being important defining factors. Our overall findings are compatible with a highly transmissible waterborne virus that is shed in urine or other secretions, and we suggest that future research should prioritize the study of potential direct transmission. The identification and spread of TFV1 in peninsular Florida provide further validation for the strict implementation of biosecurity practices in order to mitigate inadvertent transfer of aquatic pathogens.

## Introduction

Chelonians (turtles and tortoises) are one of the most threatened large vertebrate groups, with nearly 52% of 360 species categorized by the IUCN (International Union for the Conservation of Nature) as vulnerable, endangered, or critically endangered [1]. As reviewed by Stanford et al. [1], habitat loss and degradation, along with overexploitation (e.g., legal and illegal harvesting for consumption, traditional medicines, and the pet trade), are the primary threats driving the population decline of many chelonian species.

Infectious diseases are not commonly listed among the reasons for population-scale imperilment of chelonians [1]. However, there are many reports of infectious agents negatively impacting wild turtle populations. Perhaps the most striking example occurred in 2015 in Bellinger River snapping turtles (*Myuchelys georgesi*), which is a geographically restricted population. A minimum population estimate of approximately 3,000 turtles was reduced to only 150 individuals (2017 and 2018 data) following a mass mortality event that was attributed to a novel nidovirus [2,3]. *Caryospora*-like organisms have been associated with mass mortality events among green turtles (*Chelonia mydas*) in Australia and the US [4]. Respiratory *Mycoplasma* infections have caused substantial morbidity and mortality in free-ranging populations of desert tortoises (*Gopherus agassizii*) and gopher tortoises (*Gopherus polyphemus*) in the US [5,6]. In the Southwestern US, several populations of mud turtles (*Kinosternon spp*.) have developed an extensive cutaneous protozoal infection (*Epistylis* spp.) that results in diminished nutritional condition and difficulty swimming [7]. A ranavirus (*Ranavirus rana1*, formerly known as *Frog virus 3*) has been documented to cause high mortality rates in localized eastern box turtle (*Terrapene c. carolina*) populations [8,9]. Overall, viruses have been poorly studied in freshwater turtles as compared to other disease-causing organisms (e.g., parasites and bacteria) [10].

Although pathogens can significantly affect the health of wild chelonian populations, mortality investigations involving free-ranging animals are inherently challenging, and it is difficult to assess the true magnitude of the effects of disease. On-the-ground logistical issues can hinder the collection of robust field data and laboratory diagnostic samples. Frequently, there is a significant delay between the time of death and carcass detection (or reporting), and recovered carcasses are frequently scavenged or too decomposed to be suitable for evaluation. This problem is amplified in hot and humid weather, such as in Florida, and in aquatic species that often sink upon death. Furthermore, sparse population data (e.g., size, density, and home range) can hamper epidemiological studies that aim to understand pathogen transmission and maintenance in a population or estimate disease prevalence [11]. Mortality events involving multiple species and that occur in varied habitats across a broad geographic range tend to further complicate field investigations and data analyses. Furthermore, novel diseases often present additional obstacles, such as issues related to the timely development of laboratory detection assays and efficient allocation of limited resources to obtain data that will inform management applications.

Turtle fraservirus 1 (TFV1) was first discovered in Florida softshell turtles (*Apalone ferox*) in peninsular Florida in 2018 [12], and it has since been taxonomically recognized as belonging to a new viral family, *Tosoviridae*, within the Phylum *Negarnaviricota* [13]. Much remains to be learned about this novel virus. In live turtles, the clinical signs of TFV1 often include respiratory and neurological abnormalities, ocular and skin lesions (e.g., sloughing, infection, and erythema), and emaciation in some species [12]. Gross lesions observed at necropsy may include exudative ulcerations of the palpebrae, oral cavity, cloaca, and genitalia [12]. Histopathological lesions vary in severity and between species; however, vascular-associated inflammation within the nervous system is a generally consistent histopathological finding [12]. The incubation period, transmission route(s), range of virus-susceptible species, and other key epidemiological factors that pertain to this disease are still unknown. Future laboratory studies may help to provide answers; however, resources for such studies are often limited and ethical considerations warrant thoughtful study design.

Because TFV1 is an emerging pathogen, it is imperative to share available information and early research findings. Our work addresses several research goals. The first goal was to make our existing data available. The second goal was to conduct exploratory analyses to evaluate potential factors associated with a turtle testing positive for TFV1. Our analyses were designed to consider analytical restrictions resulting from resource limitations (e.g., staffing and funding) and logistical challenges (e.g., carcass scavenging and post-mortem decomposition). Third, using the results of our exploratory analyses, we wanted to summarize our findings into Florida-specific guidelines for TFV1 continued surveillance and management actions. Lastly, we used our data to make suggestions for future research.

## Methods

### Morbidity and mortality reporting and investigations

The Florida Fish and Wildlife Conservation Commission (FWC) first received reports of sick and dead Florida softshell turtles (*Apalone ferox*) in the Tosohatchee Wildlife Management Area of the St. Johns River in March 2018 [12]. Since then, the FWC has employed a variety of evolving methods to solicit, receive, and respond to reports of sick and dead freshwater turtle species across the state. The method by which each collected turtle was first reported to the FWC, termed the primary reporting method, was recorded.

Initially, an agency mechanism for reporting sick and dead freshwater turtles was not in place; reports were received by either the Chronic Wasting Disease (CWD) or the Fish Kill Hotlines (https://myfwc.com/research/saltwater/health/fish-kills-hotline/), the latter of which offered both an online reporting and call-in option. In September 2018, a designated phone number (called the Turtle Hotline) was established. Concurrently, a freshwater turtle reporting option was added to the FWC Reporter, a comprehensive mobile application launched in February 2017 to facilitate public reporting of fish and wildlife concerns to the FWC and to direct the user to the appropriate office [14]. Following a series of prompts, users of the App could choose to submit information via an emailed report and/or to select the “call a biologist” option, using the turtle hotline number. The turtle hotline and FWC Reporter were promoted through FWC media releases, informational handouts, and flyers, which were posted at >100 publicly accessible boat ramps along the St. Johns River during January and February of 2019. All of the above-mentioned passive reporting options remained available for public use through September 2021. In 2018 and 2019, the FWC performed airboat surveys and active outreach in areas where infected turtles had been detected and to stakeholders (e.g., airboat ecotourism operators) who were more likely to encounter sick and dead turtles; airboat surveys could not be conducted in 2020 and 2021 due to the COVID-19 pandemic. For further awareness, in October 2019, the FWC distributed a letter to Florida state-permitted wildlife rehabilitation centers informing them of the emerging disease in freshwater turtles, with a description of the clinical signs to look for and how to collect and submit samples from, or carcasses of, suspected cases.

Upon receiving a report, to the greatest extent possible, data were collected from the reporting party (the “reporter”) through guided conversations over the phone, which generally occurred within 24-48 hours of receiving the report. These conversations were especially valuable in cases for which site investigations could not be performed (i.e., due to delayed reporting or logistical challenges). The reporters were prompted to describe when they first observed sick or dead turtles, the number and species involved, and whether any apparently healthy turtles remained in the environment. When provided, photos shared by the reporters aided in freshwater turtle species identification and assessment of post-mortem condition. We also inquired if any other wildlife mortalities (e.g., fish) were observed during the same timeframe. In addition, if the event being reported occurred at an urban retention pond, reporters were asked if the pond had a functioning aeration system and if any changes in water quality (e.g., algal growth) had been noted. These latter three questions were designed to understand if toxins (e.g., harmful algal blooms, botulism, or chemical treatments) might have been a cause of mortality.

Turtles were acquired for necropsy by several means. Following reports, when feasible, FWC staff performed field site investigations to verify information received from the reporters and collect carcasses for necropsy. During these investigations, sick turtles were occasionally captured and transported to the FWC Wildlife Health Laboratory for euthanasia (see below). In addition to field-collections, euthanized turtles were also received directly from wildlife rehabilitation centers. Prior to euthanasia, observations of behavior were recorded when possible.

### Ethics statement

Field investigations were performed by FWC staff; thus, permitting was not required by the State of Florida for this study. When necessary, sick turtles examined by FWC veterinarians were humanely euthanized following the previously described methods [12], which were in accordance with euthanasia guidelines established by the American Veterinary Medical Association [15]. Florida state-permitted wildlife rehabilitation centers operate under rules outlined in the Florida Administrative Code 68A-9.006, which includes permitting for humane euthanasia.

### Necropsy and laboratory diagnostics

This study included 117 freshwater turtles collected from 9 March 2018 until 5 September 2021; 80 were collected deceased and 37 were euthanized. Turtle species consisted of Florida softshells (hereafter referred to as softshells), and the following, collectively referred to as hardshells: Florida red-bellied cooters (*Pseudemys nelsoni*), peninsula cooters (*P. peninsularis*), red-eared sliders (*Trachemys scripta elegans*), yellow-bellied sliders (*T. scripta scripta*), and slider hybrids. Necropsy methods followed those previously described in Waltzek et al. [12], and we incorporated 24 turtles listed in Table 2 of that initial investigation as a subset of the 117 necropsy cases analyzed in this work. Samples of major organs and gross lesions were collected from 93 turtles; the remaining 24 turtles did not have suitable tissues for testing due to post-mortem decomposition or severe scavenging.

**Fig 1:**
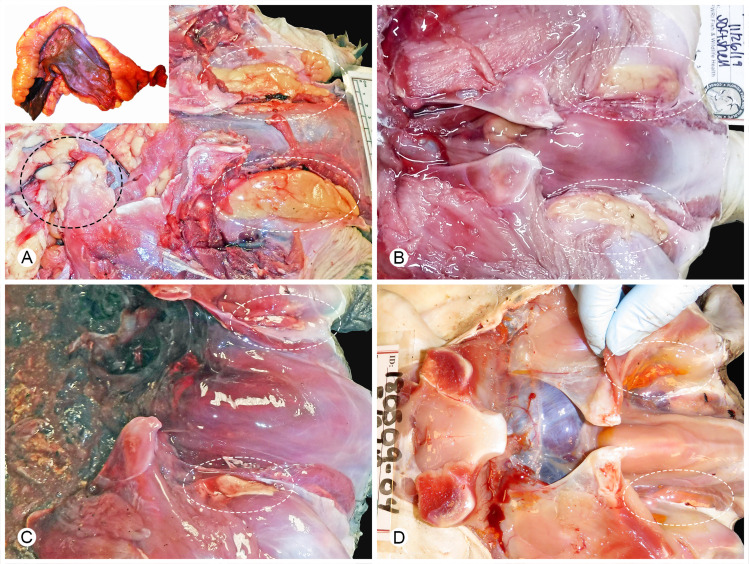
Subjective categorization of adipose (fat) tissue in Florida softshell turtles (*Apalone ferox*) evaluated at necropsy, with example images highlighting the axillary fat pad deposits (white dashed ovals). The head is oriented to the right in all images. (A) Abundant: Axillary fat is prominent, there is a visible band of fat (black dashed oval) between the plastron and the coelomic membrane, and a thick band of mesenteric fat follows the greater curvature of the stomach (inset) and intestine (not pictured). (B) Moderate: Axillary fat remains prominent, but the turtle lacks fat overlying the coelomic membrane, and mesenteric fat near the stomach and within intestine (not pictured) is less prominent. (C) Slight: Axillary fat is reduced, minimal mesenteric fat is visible on the stomach and is absent along the intestine. (D) Atrophy: Fat has a gelatinous texture and is orange-tinged.

**Fig 2:**
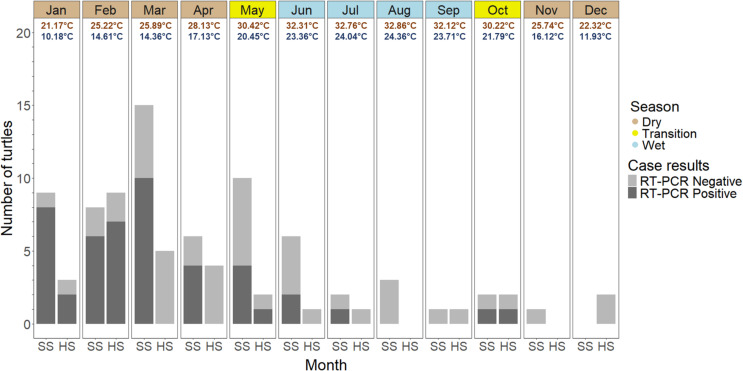
Turtles (*n* **=** **93) tested for Turtle fraservirus 1 (TFV1) via RT-PCR from 9 March 2018 until 5 September 2021 by month (top panels) and species (*x*-axis; SS** **=** **softshell, HS** **=** **hardshells).** Positive and negative cases are represented by dark and light gray bars, respectively. The monthly columns are color-coded to represent the wet months (June – September; blue), the dry months (November – April; brown), and the transitional months (May and October; yellow). The four-year average *xTmax* and *xTmin* values are included for each month, derived as described in the manuscript text.

**Fig 3:**
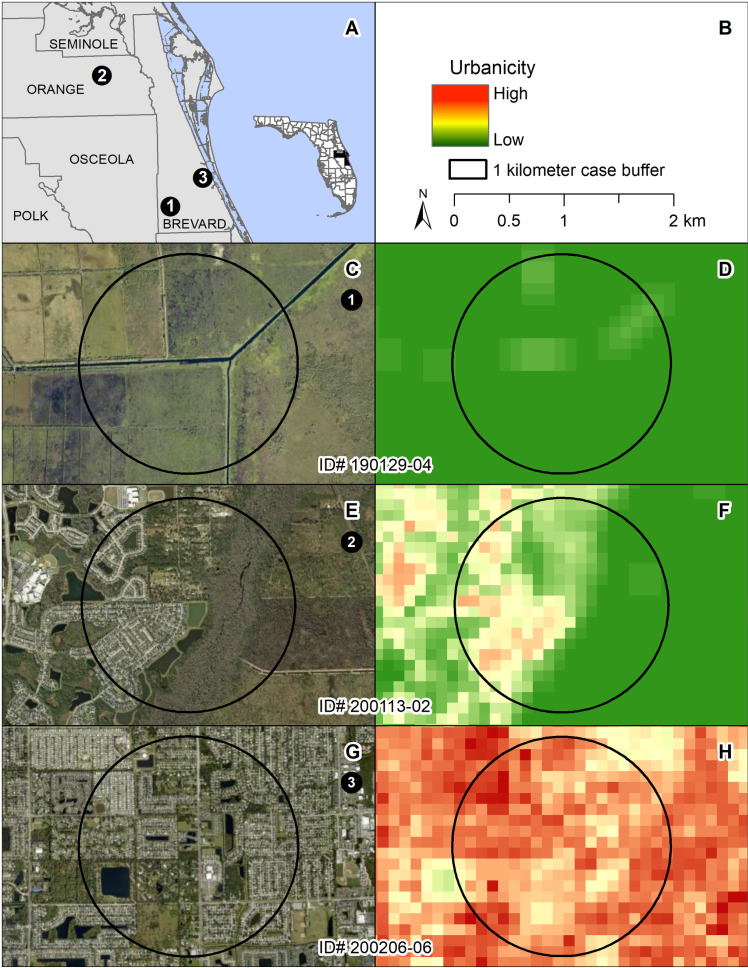
Combined urban gradient layers generated to create the *Urbanicity* variable used in the Field dataset analysis for three example turtles. Each circle represents a 1-km buffer around a turtle’s collection location. The base aerial images on the left are shown as correlates to the urban gradient layer mapping images on the right. (A) Map of Florida counties showing the locations of the three example turtles. (B) Legend depicting urban gradient from High (red) to Low (green). (C, D) Turtle 190129-04, collected from a Low Urbanicity category. (E, F) Turtle 200113-02, collected from a Medium Urbanicity category. (G, H) Turtle 200206-06, collected from a High Urbanicity category.

We created an *Urbanicity* variable as a proxy for unevaluated environmental factors that might influence infection status (e.g., effects of the levels of water pollutants on host immune system [31]). To derive this variable, we first obtained four measures of urbanicity from the specified data sets: road density [32], housing density [33], percent developed impervious surfaces [34], and percent urban cover [35]. Next, within each of these four datasets, we standardized 100 m^2^ rasters so that the values ranged from 0‒100. We then summed these raster values across the four datasets to generate a combined urban gradient layer. Natural breaks (jenks) in the raster scores of this combined layer allowed us to group the individual raster values into three urbanicity categories: low (0‒63), medium (>63‒164), and high (>164‒398). For each turtle, we calculated an urban gradient value based on the average of all rasters within a 1-km radius buffer of the turtle’s collection location. We assigned an *Urbanicity* category of low, medium, or high based on the breakdown stated above. Fig 3 depicts examples of the three urbanization divisions overlaying aerial imagery [36] to further aid field observers in categorizing future samples.

**Table 1:** Primary reporting methods (PRM) for 117 freshwater turtles collected and necropsied from 9 March 2018 until 5 September 2021. The values in parentheses indicate the number of turtles in each cell that were suitable for testing via PCR for Turtle fraservirus 1 (TFV1). App = FWC Reporter App (this reporting option was added in September 2018); TH = Turtle Hotline (this number was established in September 2018); CWD/FKH = combination of reports via the long-established Chronic Wasting Disease (CWD) and Fish Kill Hotline (FKH; this included online and call-in reporting); Rehab = wildlife rehabilitation centers (first notified of TFV1 via letter from FWC in 2019); O & S = cases received via outreach and survey efforts (river surveys were not performed in 2020 and 2021 due to the COVID-19 pandemic). One turtle collected by FWC in 2020 as opportunistic roadkill is included within the O & S category.

PRM	2018	2019	2020	2021	Total
App	0	5 (3)	3 (3)	1 (1)	9 (7)
TH	0	7 (6)	31 (22)	3 (3)	41 (31)
CWD/FKH	9 (3)	4 (2)	7 (5)	0	20 (10)
Rehab	n/a	4 (4)	13 (13)	7 (7)	24 (24)
O & S	13 (11)	9 (9)	1 (1)	n/a	23 (21)
**Total**	22 (14)	29 (24)	55 (44)	11 (11)	117 (93)

**Table 2:** Selected descriptive data for 93 freshwater turtles in Florida, USA, that were necropsied and tested via PCR for Turtle fraservirus 1 (TFV1) from 9 March 2018 until 5 September 2021. Softshells are limited to Florida softshells (*Apalone ferox*). Hardshells are the combined group of *P. peninsularis*, *P. nelsoni*, *T. scripta scripta*, *T. scripta elegans* (and hybrids). Variables marked with ^*^ were used directly in the Necropsy Model analyses, whereas those with ^†^ were used to derive the *AnyPlaques* variable, as described in the statistical methods. The *Season* variable was used in the Field Model. When variable values could not be assigned, a category of “unknown” was included. The variable female reproductive state represents the 65 female turtles included in the dataset.

	Softshells		Hardshells	
Descriptive variable	Positive	Negative	Positive	Negative
Maturity*				
Adult	34	19	11	18
Subadult	2	8	0	1
Sex*				
Female	25	20	7	13
Male	11	7	4	6
**Female reproductive state**				
Shelled eggs	6	6	0	2
Vitellogenic	12	3	7	5
Undeveloped follicles	2	5	0	1
Unknown	5	6	0	5
**Cloacal plaques present** ^†^				
No	7	24	10	12
Yes	21	1	1	2
Unknown	8	2	0	5
**Oral plaques present** ^†^				
No	6	19	8	18
Yes	26	3	3	0
Unknown	4	5	0	1
**Recently ingested food**				
No	26	14	9	14
Yes	3	7	2	3
Unknown	7	6	0	2
**Adipose**				
Abundant/Moderate	32	20	0	3
None/Atrophied/Slight	2	5	11	16
Unknown	2	2	0	0
**Carcass frozen**				
No	16	3	5	5
Yes	20	24	6	14
**Post-mortem decomposition**				
None/Minor	24	13	11	13
Moderate	2	2	0	1
Severe	10	12	0	5
**Season**				
Wet	6	10	2	4
Dry	30	17	9	15

We summarized descriptive data collected at necropsy for softshells and hardshells by TFV1 infection status. Recorded necropsy data included: collection date and location, species, intact or partial carcass weight, maturity (adult, sexually mature; subadult, not sexually mature), sex (female, male), female reproductive status (i.e., vitellogenic ovaries, shelled eggs, or undeveloped follicles), morphometrics (i.e., curved carapace length, plastron length), and gross lesions noted (e.g., oral and/or cloacal plaques). Turtles were categorized as adults based on their morphometric measurements [16,17], whether developed follicles or shelled eggs were observed in females, whether developed testes were observed in males, and the detection of spermatozoa in males when histology was available. For 11 softshell turtles in which the sex and maturity could not be assigned by the presence of reproductive organs, these individual data points were determined through a combination of the length of the tail relative to the posterior margin of the carapace and morphometric measurements [16–18]. In addition, we recorded the presence of recently ingested food (i.e., food items within the oral cavity, esophagus, or stomach), as an indicator of feeding activity. When possible, adipose tissue was subjectively scored as abundant, moderate, or slight/atrophied/none, based on the location and quantity of body fat, as described in Fig 1.

We also recorded whether the carcass had been frozen prior to necropsy and tissue collection, and we subjectively evaluated the post-mortem condition (a combination of autolysis and decomposition) using project-specific criteria. Carcasses were rated as severely decomposed if a combination of the following findings were observed: nails sloughed with minimal tension applied, normal creamy-white softshell skin diffusely discolored bluish-green, turtles bloated with gas, and visceral organs either not easily discernible or extremely friable (e.g., liver tissue fell apart when manipulated). Carcasses were rated as moderately decomposed if all visceral organs were still recognizable and most organs were not yet friable, but tissue discoloration (e.g., darkened liver) was present. Euthanized turtles (n=37) were generally recorded as having minimal to no decomposition or autolysis since necropsy occurred after euthanasia, or the animal was preserved at 4°C after euthanasia until necropsy. We performed ancillary analyses on these non-biological covariates in relation to PCR test status (S1 Text).

Initially, samples were tested using a reverse transcription conventional PCR (RT-cPCR) assay, which was developed as previously described [12]. However, testing later primarily shifted to a more sensitive reverse transcription TaqMan Quantitative PCR (RT-qPCR) assay, which targets the RNA-dependent RNA polymerase gene and has a limit of detection (analytical sensitivity) of 102 viral copies [19]. All RT-qPCR assays were carried out in triplicate using TaqMan™ Fast Virus 1-Step Master Mix (Applied Biosystems) in 20 μL volumes containing 0.9 μM of each primer (TFV-F [5`-GCAATTGCCGCACGATTT-3`] and TFV-R [5`-TCCAGCAATGACCATGTTCCT-3`]), 0.25 μM of probe (TFV-P [5’-TGTGTCAGATTGCAGAACA-3’]), 4 μL of nucleic acid template, 5 μL of 4× universal RT-qPCR mix, and 8 μL of molecular-grade water. Reactions were carried out on a QuantStudio 5 Real-Time PCR System (Applied Biosystems) using the fast protocol thermocycling conditions: 50°C for 5 min and 95°C for 20 s; followed by 40 cycles at 95°C for 3 s and 60°C for 30 s. The result was interpreted as TFV1-positive if the cycle threshold (Ct) calculated from the 6-carboxy-X-rhodamine (ROX)-normalized 6-carboxy-fluorescein (FAM) signal in at least two of the three replicate wells exceeded the threshold automatically assigned by the Applied Biosystems software. A turtle was classified as TFV1-positive if one or more samples submitted from the same individual tested positive via either RT-cPCR or RT-qPCR.

The type and number of samples submitted for each turtle for molecular diagnostics varied according to post-mortem condition, tissue availability, and financial resources. Based on the results obtained in the first year of the investigation [12], kidney, liver, brain, and spleen were prioritized for PCR. For turtles in poor post-mortem condition, the kidney was most frequently selected because this organ often appeared to be less decomposed than other tissues on gross examination. To conserve financial resources, two tissues were occasionally pooled for testing. For twenty turtles, swabs (cloacal, oropharyngeal) and/or urine were tested in addition to tissues, and for nine turtles, swabs and/or urine were the only samples tested. To reduce the likelihood of false-negative outcomes, turtles in poor post-mortem condition that tested negative on RT-cPCR were retested with the more sensitive RT-qPCR if the necropsy did not reveal an obvious cause-of-death or if the turtle was collected from a geographic area with confirmed positive turtles. Based on history, necropsy findings, and occasionally histopathology or ancillary diagnostics, the cause-of-death for these negative cases was categorized as suspected TFV1-false-negative, other infectious causes (non-TFV1), trauma, toxins, or unknown cause.

Due to limited funding, formalin-fixed tissues were only submitted from approximately one-third of the turtles necropsied in this study. The initial investigation [12] primarily summarized TFV1-associated histopathological lesions in softshell turtles, and we therefore prioritized additional histopathology on a case-by-case basis to non-softshell species and unusual findings observed on gross necropsy. However, we do not incorporate histopathological findings into our statistical modeling (discussed below).

### Statistical analyses

We used logistic regression to determine the influence of recorded explanatory variables on the probability of TFV1-positive status. In 2018, 2019 and 2020, if samples could be collected, all necropsied softshells and hardshells were tested for TFV1 and included in our analyses, regardless of carcass post-mortem condition. In 2021, FWC prioritized resources such that only carcasses with minimal to no decomposition and autolysis were necropsied and tested; turtles collected up to 5 September 2021 were included in the analyses. Data were acquired from field data collection, necropsy findings, weather information, and analysis of surrounding spatial patterns. Variables used in the statistical analyses were either collected during sampling or were derived based on *a priori* hypotheses of factors that may influence the probability of a turtle testing TFV1-positive.

Four datasets (**Field**, **Necropsy**, **Weather**, and **Spatial**) were created to evaluate the data available from different stages of a mortality investigation, since decisions regarding TFV1 investigation efforts may be dependent upon resource capacity. All analyses were completed using R statistical software, version 4.1.3 [20]. Due to sample size limitations, no more than five parameters were estimated in any model [21]. Within each dataset, Akaike’s information criterion, adjusted for small sample sizes (AIC_c_ and ΔAIC_c_; [22]), and Akaike weights (W_i_, [23]) were used to determine the most plausible models, or the models that best characterized the variation in the probability of a TFV1-positive PCR assay. We considered all models with ΔAIC_c_ <4.00 as having strong support [23,24]. We evaluated the predictive performance of each model by assessing the area under the receiver operating characteristic curve (AUROC) and Brier score using the ROCR and DescTools packages, respectively [25, 26]. An AUROC value of 0.50 indicates discrimination of the TFV1 status is based on random chance, whereas an AUROC value of 1.0 denotes perfect discrimination of TFV1 status. Brier scores measure the accuracy of probabilistic predictions, where zero values represent complete accuracy and scores of 1.0 signify fully inaccurate predictions. All inferences were based on the best-approximating models for each dataset, and within the supported models, predictor variables were considered informative if the 95% confidence interval of the associated coefficient did not overlap zero. Lastly, for each dataset, we fit a global model with no interaction terms to assess evidence of collinearity and excluded all variables with a variance inflation factor (VIF) >5 [27]. The candidate sets of models were also assessed for model fit through residual diagnostics on scaled residuals using a simulation approach with the DHARMa package [28].

The **Field** dataset included four variables (*CL7*, *Species*, *Season*, and *Urbanicity*) that could be derived without a hands-on examination of sick or deceased turtles, with little knowledge of geographically related turtle morbidity and mortality events, and by anyone who reported field observations (e.g., including wildlife rescue groups, public citizens, FWC personnel). We defined *CL7* as a categorical response (yes or no) to represent a simple cluster status based upon the collection date and location of each necropsied turtle. A “yes” value was assigned to the necropsied turtle if both of the following conditions were met: 1) at least one other sick or dead turtle, of any species, was observed within a 7-day period of the necropsied turtle’s collection date, and 2) the observed morbidities/mortalities occurred at the same defined body of water (e.g., urban retention pond, natural lake, marsh) or within the system of connected canals in Palm Bay, as described by Waltzek et al. [12]. We included *CL7* in the Field dataset because we suspected that a sick or dead turtle would have a higher probability of testing positive for TFV1 if it was closely related in space and time to other sick and dead turtles. It is important to emphasize that categorization of the *CL7* variable did not require the collection of turtles for sampling and testing, and thus this variable could be evaluated in situations where testing was not feasible. The *Species* variable was set as a categorical variable denoting if a turtle was a “hardshell” or “softshell” for two reasons. First, it can be difficult for public citizens and others reporting field observations to differentiate cooter and slider species, whereas these hardshells can be easily discerned from softshells. Second, our dataset contained relatively low numbers of each individual hardshell species. The interaction term between *CL7* and *Species* was evaluated to determine if the odds of a species group testing positive for TFV1 would differ depending on *CL7* status.

Given that many wildlife diseases exhibit seasonal trends [29], we incorporated a categorical *Season* variable as a coarse representation of the time of year and weather patterns. Based on Florida weather analyses [30], turtles were categorized as being collected during either the Wet (May 15^th^ to October 14th) or Dry (October 15th to May 14th) seasons. This dichotomous variable could be easily assigned based on the month and without the need for data mining (e.g., gathering weather data) from online resources. Our sample size was not large enough to allow us to include collection month as a covariate in our Field dataset models. Therefore, as an ancillary and more granular approach to our temporal analysis, we tallied the TFV1 infection status for softshells versus hardshells by month across all years (Fig 2).

The **Necropsy** dataset included five variables (*Species*, *Sex*, *Maturity*, *AnyPlaques*, and *Plastron Length*-*PL*) gathered from data collected by the experienced personnel who performed necropsies on the collected turtles. These data could therefore only be obtained when there were no logistical constraints that might preclude the retrieval of carcasses for necropsy, such as remote or inaccessible field locations, delayed reporting of mortalities, or unavailability of agency personnel to respond to a field report. The presence of cloacal and oropharyngeal plaques, which were frequently observed in the TFV1-positive cases described by Waltzek et al. [12], were assessed in combination: a turtle was categorized as “yes” for the variable *AnyPlaques* if cloacal, oral, or both types of plaques were present, and as “no” if no plaques were observed. For softshell turtles, we used Pearson’s chi-square test for independence between oral and cloacal plaques. We found that the presence of oral and cloacal plaques was significantly associated (χ^2^ (1, *N* = 49) = 18.00, *P* < 0.001), and that a mismatch between the presence of plaques in both locations occurred in only 18% of cases. Thus, to retain available softshell data, if either the oral or cloacal region could not be evaluated (i.e., one region was missing or severely autolyzed), we assumed that the absence of plaques in one anatomical location indicated the absence of plaques in the unevaluated location (*n* = 2). However, we could not make this same assumption for hardshells because earlier results suggested that this group of turtles did not present with cloacal plaques [12]. Therefore, in hardshells, if one anatomical location could not be evaluated and plaques were not observed in the evaluated region, then the *AnyPlaques* value was classified as unknown (*n* = 5), and these turtles were removed from further analysis within the necropsy dataset.

We included a continuous variable of plastron length (*PL*; cm) to evaluate whether the size of the turtle would influence the probability of testing positive for TFV1, perhaps through the surface area available to potential blood-feeding vectors [37], influences on movement and home range size [38], or basking behavior [39]. Although carapace measurements are routinely evaluated for many turtle species, we chose to evaluate the *PL* instead for two reasons. First, in softshells the margins of the carapace frequently curl and distort on carcasses exposed to sun and heat; therefore, measuring the rigid *PL* provides a more reliable approach for varying post-mortem conditions across carcasses. Secondly, we wanted to be consistent with previously published studies on Florida softshells that assessed sexual maturity relative to *PL* [17,40]. The *PL* was not initially measured in necropsied turtles, and the analyzed data was therefore a combination of true measurements and predicted lengths. To account for missing data in Florida softshells, we first performed linear regression to estimate *PL* based on curved carapace length (CCL; cm), a measurement that was routinely collected. Curved carapace length (t_60_ = 45.60, *P* < 0.001) described 97% of the total variation in plastron length (R^2^ = 0.97). The linear equation: Plastron length = 2.00 + 0.69 * Curved carapace length (cm), was used to impute missing plastron lengths for six turtles with available carapace length data. For five additional softshell turtles that were also lacking CCL data, we fitted a weight-length log-linear regression to 45 softshell turtles within the dataset with available measured plastron lengths and intact weights. Log-weight (g) described 94% of the total variation in log-plastron length (t_43_ = 25.65, *P* < 0.001). The equation *Plastron length (cm) = e*^0.87 + log(weight(cm)) * 0.30^ was therefore used to predict and impute plastron lengths for these five softshell turtles. Two hardshells turtles (peninsula cooters) with missing plastron lengths were removed from the dataset. We did not predict plastron lengths for this hardshell turtle species as we were limited by the available sample size (n=18 peninsula cooters).

The **Weather** dataset incorporated variables that represented temporally and spatially associated weather data. We assumed that these variables would influence potential vector ecology (e.g., mosquito abundance) and turtle physiology (e.g., reproductive activity and brumation), which, in turn, could influence disease. Turtle location and collection date were used to collate data for the following four continuous variables: day-length (*DL*; hours), average maximum temperature (*xTmax*), average minimum temperature (*xTmin*), and average precipitation (*Precip*). We obtained the day-length for the turtle collection date and location using the R package “insol”, which calculates the day-length based on the latitude, longitude, Julian day, and time zone [41]. The remaining weather data were evaluated across a 30-day interval (turtle collection date + 29 days prior). This decision was influenced by our necropsy observations that fat stores were not depleted in TFV1-positive softshells, suggesting that the disease process, relative to turtle metabolism, was unlikely chronic. Furthermore, in a few mortality events, clusters of sick and dead turtles were observed at a single body of water over a minimum duration of three to four weeks. Thus, it is possible that after the virus is locally introduced, transmission and incubation (time from infection to development of clinical signs) could occur over a period of several weeks. Finally, although the incubation period for TFV1 is unknown and likely varies by species, published literature on other viruses that cause turtle mortalities have shown that severe clinical signs often develop between 6 to 31 days post-inoculation, with survival times varying by species, age, virus exposure routes, and ambient temperature [42–44].

We extracted precipitation and temperature data from two online sources. We primarily acquired precipitation data from the CoCoRaHS (Community Collaborative Rain, Hail, and Snow Network; https://www.cocorahs.org/) database, as described in the S1 Text and S1 Appendix. For each turtle, precipitation data from all site-specific CoCoRaHS stations (within a 16-km radius of the turtle’s collection location) were summed across the 30-day period and then averaged (data available in S1 Table). We used this localized CoCoRaHS precipitation data in the weather dataset models for all but seven turtles, for which data were unavailable. For these seven cases, we obtained precipitation data from the closest National Oceanic and Atmospheric Administration (NOAA) station for each turtle; thus, the precipitation value is the sum of the recorded precipitation across the 30-day period. Our NOAA temperature data acquisition methods are described in detail in the S1 Text and in the S2 Appendix. In brief, we viewed the Florida mapped NOAA weather stations (https://www.ncei.noaa.gov/maps/daily-summaries/), pulled up relevant station details and downloaded historical weather data. For each turtle, the NOAA weather station closest to, but no greater than, 60 km (mean = 18 km) from the turtle’s collection location (latitude/longitude) was identified. The VIFs for *xTmax* and *xTmin* exceeded the selected threshold, so the respective variables were not included within the same models. We used temperature data from these same identified NOAA stations (S1 Table, column NOAA Stn ID) to derive four-year (2018–2021) average *Tmax* and *Tmin* values for each month and present these data in Fig 2.

We formed a **Spatial** dataset to evaluate spatial and temporal virus patterns beyond the conservative *CL7* variable included in the Field dataset. This analysis was structured to capture general trends in the probability of a turtle testing positive for the virus given that turtle’s relationship to other TFV1-positive turtles. Least-cost-paths (LCP) were created to relate the proximity of all turtles with PCR results (see S1 Text). For each turtle observation in the dataset (the *Target* turtle), all TFV1-positive turtles located within a radius of 25 km were categorized as *Associated* turtles. We selected four mutually exclusive cluster categories to describe the LCP distance between target and associated turtles: a tight cluster (*Tight*) of 0.0–1.0 km, a loose cluster (*Loose*) of 1.1–5.0 km, a distant cluster (*Distant*) of 5.1–10.0 km, and a possible cluster (*Possible*) of 10.1–25.0 km. These distances were based on movement studies of various species of softshell turtles, which reported linear home ranges (the distance between the furthest upstream and downstream recorded locations [45]) of less than 1 km to nearly 87 km [18, 46, 47]. For each *target* turtle, the sum of *associated* TFV1-positive turtles within each of the four cluster categories was calculated and used as a continuous variable value in the model. A second set of variables was created following the same clustering process, but each category was truncated to only include TFV1-positive turtles that were collected within ±30 days of the *target* turtle’s collection date (*Tight30*, *Loose30*, *Distant30*, and *Possible30*). We considered 30 days in either direction (±30 days) because we were unable to assess temporal directionality (e.g., the significance of Turtle A being detected TFV1-positive before Turtle B, or vice-versa) since positive spatially and temporally related turtles may have gone undetected with our method of convenience sampling. We fitted two models to the data in which each set of clusters (no time component and ±30-day clusters) were continuous explanatory variables. We included only additive combinations of each cluster as we had no biological hypothesis to support interactive effects. We did not use model selection methods on the Spatial dataset and instead report the results of both models because we were interested in the relevant patterns for each set of variables.

## Results

### Morbidity and mortality reporting and investigations

The primary reporting methods by which all turtles were obtained for necropsy (*n* = 117) are presented in Table 1. The number of reports received via the Chronic Wasting Disease (CWD) and Fish Kill hotlines decreased between 2018 and 2021 as other taxa-specific reporting methods were established and repeatedly promoted. Use of the FWC Reporter App varied across the years. Overall, 7.7% (9/117) of the necropsy cases were reported via the App as emails, and fewer reports were received each year via this method as opposed to the Turtle Hotline. However, it is possible that some of the App reports were redirected to the turtle hotline if an App user decided to select the “call a biologist” option rather than making the final App submission via email. Carcass suitability for PCR testing was approximately equal for reports received via the App and the Turtle Hotline, from which 77.7% and 75.6% of collected turtles were tested, respectively. The percentage of direct reports from wildlife rehabilitation centers increased over the years and represented >63% (7/11) of cases in 2021, reflecting the working relationships that were developed between these centers and FWC wildlife health team members. All 24 carcasses received from these centers were suitable for PCR testing since the turtles were chemically euthanized or died spontaneously while under care. Before euthanasia, all turtles displayed lethargy and minimal response to stimulation. Additional antemortem observations (e.g., aural abscess, respiratory distress) are reported in S1 Table.

### Necropsy and laboratory diagnostics

We necropsied 117 turtles, of which 93 (79.5%) were tested for TFV1 by PCR on one or more samples. We found that 57.1% (36/63) of softshells and 36.7% (11/30) of hardshells tested positive for TFV1. Microscopic lesions associated with TFV1 infection required histopathology for detection and were similar to those previously described [12]. Moderate or severe meningoencephalitis with and without vasculitis in other organ systems were consistent observations among infected softshell turtles, whereas meningitis observed in hardshells was relatively mild.

Table 2 presents the descriptive data for the necropsied turtles across all years, by species group and test status. Due to factors such as decomposition and scavenging, recently ingested food items were recorded as *unknown* for 32/117 of all necropsied turtles and for 15/93 turtles tested by PCR. Excluding the *unknown* turtles, food items were observed in 33.3% (7/21) of TFV1-negative softshells but in only 10.3% (3/29) of TFV1-positive softshells. In contrast, for hardshell species, food items were observed with similar percentages for TFV1-negative (17.6%) and TFV1-positive turtles (18.2%). For turtles in which fat could be evaluated, <12% of softshells did not have moderate to abundant fat, whereas 90% of hardshells were lacking fat stores, regardless of virus status (Table 2).

A total of 216 samples from 93 turtles were tested by RT-PCR, of which kidney (*n* = 61), liver (*n* = 41), brain (*n* = 31), and spleen (*n* = 21) comprised the majority of samples (see S1 Table for results by sample type). The number of samples tested from each turtle ranged from one to eight, with a median of two samples per animal. For nine turtles, a pooled sample of two tissues, generally kidney and liver, was the only submission. Urine samples proved to be reliable for testing. Specifically, 4/4 urine samples tested positive from turtles that also had positive tissue (e.g., liver, kidney, brain, spleen) results, and two additional urine samples tested positive from turtles with positive cloacal and oral swabs but no tissue results. For every turtle, the PCR results were consistent across all submitted tissue sample types (i.e., all were negative, or all were positive). However, five softshells and one peninsula cooter had a negative RT-cPCR assay, but a positive RT-qPCR assay. Some of the discrepancies between these assays were likely a result of samples with low viral loads, which were probably below the analytical sensitivity of the RT-cPCR assay.

The morbidity conditions observed in the 46 TVF1-negative turtles are listed in S1 Table. The cause-of-death of 18 TFV1-negative turtles was considered unknown. The remaining 28 turtles were categorized as trauma (*n* = 19; e.g., vehicular/boat, predation, conspecific, fishhook), infections (*n* = 3; e.g., pneumonia, renal, fungal), neoplasia (*n* = 1), suspected toxin exposure (*n* = 1), and suspected TVF1 false-negatives (n=4). All of the suspected false-negatives originated from clustered mortality events (*CL7* = yes). The head was missing from one softshell (200706-01); however, cloacal plaques were observed in this animal, and also in another softshell from this same mortality event that was necropsied but not tested (200706-02). In a separate mortality event in which three softshells were collected on the same day, cloacal and oral plaques were not observed in two turtles (200729-01 and -02), but the third (200729-03) tested positive with discrepant RT-cPCR and RT-qPCR results. Finally, one of the first turtles collected from the St. Johns River during the initial mortality investigation (180313-02) was retained in a freezer for more than a year prior to necropsy. All seven other softshells collected and tested from this region tested positive, and thus it is possible that prolonged freezing affected the detection of the viral nucleic acid. As described in more detail in the S1 Text, in our ancillary analyses, we found that turtles that were frozen prior to necropsy were less likely to test positive for TFV1, but that post-mortem condition was not associated with TFV1 test results.

### Model outcomes

The complete set of data used in the analyses is available in S1 Table. Due to missing data, each logistic regression dataset included a varied number of turtles. The full results of the models for each of the first three datasets are provided in the associated supporting table (S2 Table), and only the top supported models (Table 3) are reported below. For all top supported models presented, the AUROCs and the Brier scores were ≥ 0.67 and ≤ 0.22, respectively.

**Table 3:** Top supported model subset outcomes for each dataset, where delta AIC value ≤4.0. Estimate and SE (standard error) represent logit scale estimates of select models. Odds ratio = exp (Estimate); *n* denotes model sample size. For categorical variables, zero served as the statistical baseline as specified: *CL7* (0 = no), *AnyPlaques* (0 = no), and *Species* (0 = hardshell). For each dataset, comparisons of all model subsets evaluated can be found in S2 Table. Area under the receiver operating characteristic curve (AUROC) and Brier scores describe model performance and prediction accuracy, where an AUROC of 1.0 is perfect and a Brier score of 0.0 is perfect.

Dataset (n)	AUROC/Brier scores	Predictor variables	Estimate	SE	Odds ratio	Odds ratio 95% CI
Field (89)	0.81/0.15	Intercept	-1.55	0.42		
		CL7 (yes)	2.91	0.55	18.39	6.65–57.60
Necropsy (81)	0.84/0.14	Intercept	-0.22	0.47		
		AnyPlaques (yes)	0.22	1.11	1.25	0.13–12.42
		Species (softshells)	-1.44	0.73	0.26	0.05–0.94
		AnyPlaques*Species	3.45	1.35	31.50	2.17–491.59
Weather (90)	0.70/0.22	Intercept	5.54	1.84		
		xTmax	-0.20	0.07	0.82	0.71–0.93
Spatial (90)	0.89/0.13	Intercept	-1.40	0.37		
		Tight	1.27	0.44	3.57	1.70–9.67
		Loose	0.75	0.27	2.12	1.32–3.89
		Distant	0.08	0.17	1.08	0.78–1.55
		Possible	0.13	0.09	1.14	0.98–1.38
	0.81/0.16	Intercept	-0.97	0.31		
		Tight30	1.61	0.48	5.00	2.19–15.51
		Loose30	0.92	0.37	2.51	1.31–5.88
		Distant30	0.16	0.25	1.17	0.74–2.07
		Possible30	0.09	0.23	1.09	0.68–1.77

**Fig 4:**
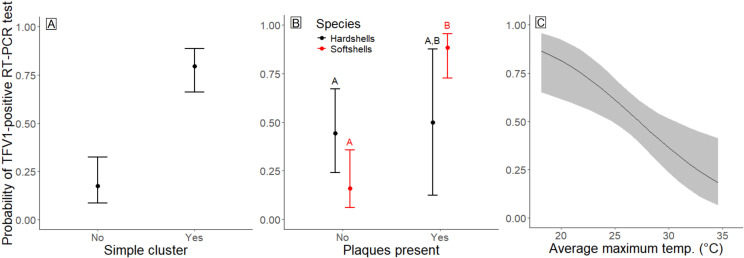
Graphical representation of the predicted probability of a turtle testing positive for Turtle fraservirus 1 (TFV1) via PCR based on significant predictors from the top supported Field, Necropsy, and Weather models. (A) Field model with simple cluster (*CL7*), (B) Necropsy model with presence of cloacal and/or oral plaques (*AnyPlaques*), and (C) Weather model with average maximum temperature (*xTmax*). Points represent average predicted probability and error bars represent 95% confidence intervals for panels A and B; lines represent average predicted probabilities and shaded regions represent 95% confidence intervals for panel C. Pairwise comparisons are significantly different (α = 0.05) when letters (A and B) cannot be matched across pairs.

The **Field** dataset contained 89 turtles (30 hardshells and 59 softshells). Within this dataset, the model that best supported the probability of a turtle testing positive contained only the simple cluster (*CL7*) variable. All models that included *CL7* also received support (S2 Table: Field). There was no support that additional predictors influenced the probability of a turtle testing positive, including the interaction between *CL7* and *Species*. Based on the estimated odds ratio, turtles that were collected from a clustered event were 18 times more likely to test positive than turtles that were not collected from a clustered event (Table 3). The probability of testing positive was 0.80 [0.66, 0.89 95% CI] and 0.18 [0.09, 0.32 95% CI] for turtles from clusters versus non-clusters, respectively (Fig 4A).

Our models did not identify *Season* as a significant predictor for TFV1 infection status. However, as seen in the frequency histogram (Fig 2), there was a general trend of an increased number of positive detections during the first quarter of the calendar year. During the months of January, February, and March, 67.3% (33/49) of the collected turtles tested positive, representing 70.2% (33/47) of the total number of positive turtles collected across the study period.

The **Necropsy** dataset contained 81 turtles (22 hardshells and 59 softshells). A single model was supported from the nine candidate models (S2 Table: Necropsy). This model contained *AnyPlaques*, *Species*, and the interaction between these two variables. Of 36 TFV1-positive softshells, 83.3% (*n* = 30) presented with *AnyPlaques* as compared to only 27.3% (*n* = 3) of 11 TFV1-positive hardshells. Although there was a significant effect of the interaction between *Species* and *AnyPlaques*, the estimate variability was large, in part because few hardshells exhibited plaques, regardless of infection status (Fig 4B). The odds ratios indicated that softshell turtles with plaques were 7.7 times more likely to test positive than hardshells with plaques, with comparative probabilities of 0.88 [0.73, 0.96 95% CI] to 0.50 [0.12, 0.88 95% CI], respectively. Furthermore, softshells with plaques were 39 times more likely to test positive than softshells without plaques, which had a probability of 0.16 [0.06, 0.36 95% CI] of testing positive. Hardshell turtles with and without plaques showed no difference in the probability of testing positive (0.50 [0.12, 0.88 95% CI] versus 0.44 [0.24, 0.67 95% CI], respectively).

The **Weather** dataset contained 90 turtles (30 hardshells and 60 softshells). Although six models were supported (*xTmax*, *xTmin, xTmax* + *DL, xTmax* + *Precip*, *xTmin* + *Precip*, and *xTmin* + *DL*), the top supported model contained *xTmax* as a single covariate. We also found similar results for the second reported model, which contained only *xTmin*. The *Precip* odds ratio and *DL* odds ratio confidence intervals overlapped 1 in the models, suggesting that these variables were not strong predictors of the probability that a turtle would test positive for the virus. In general, higher temperatures were associated with a lower probability of a turtle testing positive (see S2 Table: Weather). For each 1°C increase in the *xTmax*, there was an 18% decrease in the odds that a turtle would test positive (Fig 4C). At the lowest *xTmax* (18.17°C; 64.72°F) that corresponded to a turtle’s collection-date 30-day interval, the probability of testing positive was 0.86 [0.65, 0.96 95% CI]. In contrast, at the highest corresponding *xTmax* (34.57°C; 94.23°F), the probability of a turtle testing positive for the virus decreased to 0.18 [0.07, 0.41 95% CI].

**Fig 5:**
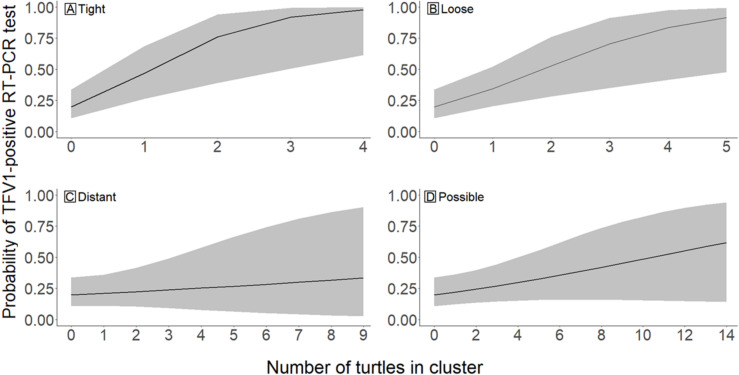
Predicted probability of a turtle testing positive for Turtle fraservirus 1 (TFV1) via RT-PCR based on the number of TFV1-positive turtles (*x*-axis) detected within a specified cluster radius. (A) Tight (0–1 km), (B) loose (>1–5 km), (C) distant (>5–10 km), and (D) possible (>10–25 km). Lines represent average predicted probability, and shaded regions represent 95% confidence intervals. Each cluster category illustrates the mean and 95% confidence interval predictions for that cluster, where the cluster categories not being explored were held constant at zero turtles.

**Fig 6:**
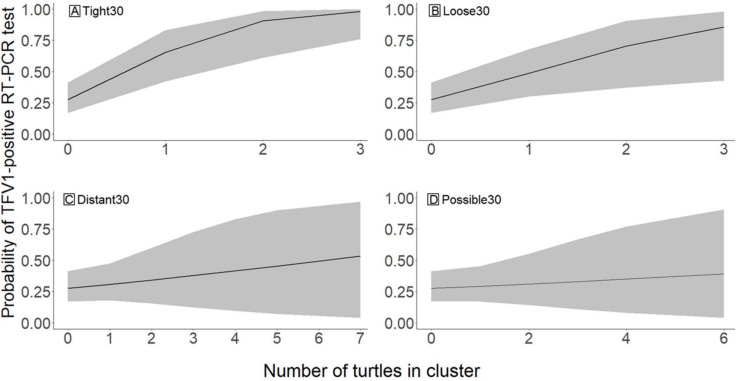
Predicted probability of a turtle testing positive for Turtle fraservirus 1 (TFV1) via RT-PCR based on the number of positive turtles (*x*-axis) detected in a **+/-30-day period and within a specified cluster radius.** (A) Tight30 (0–1 km), (B) Loose30 (>1–5 km), (C) Distant30 (>5–10 km), and (D) Possible30 (>10–25 km). Lines represent average predicted probabilities, and shaded regions represent 95% confidence intervals. Each cluster category illustrates the mean and 95% confidence interval predictions, with the remaining cluster categories held constant at zero turtles.

The **Spatial** dataset contained 90 turtles (30 hardshells and 60 softshells). Both time and the distance between the *target* turtle and *associated* positive turtles influenced the probability of a turtle testing positive for the virus. Turtles that were geographically closer to higher numbers of positive turtles had a higher probability of testing positive for the virus. Additionally, turtles that were geographically and temporally (±30 days) closer to higher numbers of infected turtles had slightly higher probabilities of testing positive than those who were only spatially clustered. The number of positive turtles in *Tight* and *Loose* cluster categories were significant predictors of a *target* turtle’s virus status, whereas *Distant* and *Possible* cluster categories were not supported predictors (Fig 5). For each additional positive turtle within the *Tight* and *Loose* cluster categories, the *target* turtle was approximately four times and twice as likely to test positive, respectively. *Tight30* and *Loose30* were also supported variables in the ±30-day time truncated model (Fig 6). *Target* turtles were five times more likely to test positive with each additional positive turtle in *Tight30* and approximately three times more likely to test positive with each additional positive turtle in *Loose30*. Confidence intervals of *Distant30* and *Possible30* overlapped zero and were not supported variables.

## Discussion

### Morbidity and mortality investigation

Turtles were obtained for this study via a passive surveillance process, which relies on the reporting, collection, and sampling of sick and dead animals [48], and thus does not allow for an estimate of pathogen prevalence within a population. We recovered carcasses for necropsy most often when turtle reports were received via the turtle hotline and from wildlife rehabilitation centers. In 2018, most turtles were collected during geographically targeted field surveys along the St. Johns River and from reports received via existing hotlines. However, after the turtle hotline was established and extensively promoted, the majority of necropsied turtles were obtained via this reporting mechanism. Reporters who contacted the FWC via the turtle hotline were generally able to provide detailed information that facilitated a rapid field response. Sometimes, these conversations generated a sustained interest in the investigation, thereby fostering continued communication and monitoring with the reporters. Building relationships with staff at wildlife rehabilitation centers proved to be crucial to our investigation, as this enabled the recovery of recently deceased turtles with minimal autolysis. In addition to providing TFV1-positive turtles, these centers were a valuable source of turtles that died of other causes (e.g., vehicular trauma, pneumonia), which allowed us to make comparisons between TFV1-positive and TFV1-negative animals.

### Necropsy and laboratory diagnostics

During the necropsy process, observations on recently ingested food and adipose could only be recorded for a subset of the turtles; therefore, these variables were not included in the Necropsy dataset models. However, these data may be informative for future investigations, since they suggest that the clinical course of disease caused by TFV1 may vary across turtle species.

The abundant-to-moderate adipose stores observed in TFV1-positive softshells indicate that the disease may have an acute course in this species. Food items were assessed for 29 TFV1-positive softshells and were only observed in three adult individuals (10.3%); contents consisted primarily of small fish bones and snail parts. In contrast, for the 21 TFV1-negative softshells for which food items were assessed, seven (33.3%) (six adults, one subadult) had recently eaten, with items consisting of meaty tissue, fish bait (fish-hook trauma case), damselflies, a small red-bellied cooter, and a rat pup (species not identified) clamped in a turtle’s mouth. Many of the negative softshell cases were presumably healthy immediately prior to death, with various causes of trauma being detected at necropsy.

It is worth mentioning that the stomach of one adult TFV1-positive softshell contained abundant sand (stomach weight of 230 grams) mixed with several blades of grass. A ruptured caseous encapsulated mass (6.3 cm × 3.0 cm) containing dirt and vegetative matter, including small roots, was observed within the large intestine of this individual. It is unknown whether this finding was due to disease-induced abnormal feeding behavior or reduced passage of sand, which may be incidentally ingested by softshell species [49,50].

The hardshells included in this study were generally sick individuals (either TFV1 or other illnesses, such as pneumonia), which would make them prone to anorexia. It is therefore reasonable that the presence of recently ingested food was similar (~18%) between TFV1-positive and -negative hardshells. Furthermore, TFV1-positive hardshells were generally lacking adipose tissue, indicating either a more chronic disease course in these species, or possibly that individuals with underlying disease (thus already in poor body condition) were more susceptible to clinical TFV1 disease.

Multiple sample types (i.e., tissues, swabs, and urine) can test positive for TFV1 via RT-PCR assays, and usually, the test results are consistent across sample types within an individual turtle. Thus, the selection of samples can be situation-specific, taking into consideration animal disposition (e.g., alive or dead), carcass condition, and carcass organ availability (e.g., some tissues may be missing due to scavenging). For field-deceased turtles with more advanced post-mortem decomposition, the kidney may be preferred over other tissues that appeared to decompose more rapidly (e.g., liver, spleen, brain) or are more difficult to extract (e.g., brain). Cloacal swabs, oral swabs and urine are valid samples for antemortem testing when turtles are displaying clinical signs of illness. Finally, because our ancillary analysis suggested that freezing may influence PCR status outcome, we suggest avoiding extended freezes of carcasses prior to necropsy and collection of tissues, as well as multiple freeze-thaw cycles of samples. However, we did not collect or analyze data in a manner that allows us to provide maximum threshold values for freeze times of carcasses and samples.

Overall, exceptional concordance was obtained for the RT-cPCR and RT-qPCR assays. Discrepant results were observed for tissues from four severely autolyzed softshells, for which liver, kidney, and brain tested negative on RT-cPCR but positive on RT-qPCR (*Ct* values ranged from 28.70 to 39.47). Three of these cases were classified as *yes* for both *AnyPlaques* and *CL7*, thus the positive RT-qPCR results align with significant predictors of TFV1. In addition, a pooled kidney and brain sample from a peninsula cooter (200824-02) was also negative on RT-cPCR and positive on RT-qPCR (*Ct* = 38.04). This turtle was found alive in the middle of a road. Upon admission to a wildlife rehabilitation center, shell abrasions and blood within the oral cavity suggestive of vehicular trauma were observed. The turtle was euthanized, and gross lesions found on necropsy confirmed vehicular trauma. In this case, the cooter was in excellent post-mortem condition, and the negative RT-cPCR was likely a result of a low viral load in this individual.

### Model outcomes

Our **Field** dataset models revealed that turtles collected from conservatively defined clustered mortality events (*CL7*) had a much higher probability of testing positive for TFV1 than turtles that were not associated with such events. This finding supports the idea of a direct route of virus transmission. A citizen who observes and reports sick and dead turtles would typically only have knowledge of events occurring over a limited time period and at a specific location, such as a neighborhood retention pond. Similarly, a local wildlife rescue group might recognize that multiple calls had been received from the same area over a short timeframe. Understanding the epidemiological importance of the simple cluster status (*CL7*) is useful towards assessing the probability of TFV1 mortalities and the subsequent prioritization of necropsy, sample collection, and laboratory testing. In situations where follow up investigations are not pursued (e.g., because of logistical challenges, lack of resources, carcasses in poor postmortem condition), knowledge of the *CL7* status, which does not require PCR test results, can help to guide conversations with local stakeholders on the importance of promptly reporting additional mortalities, documenting clinical signs observed in live animals, etc.

Waltzek et al. [12] suggested vector inoculation may be one route of TFV1 transmission. Leeches were commonly observed on the turtles in this study, but only two leeches were tested for TFV1 from a single positive peninsula cooter (190226-02), one of which was positive. However, the significance of this finding is unknown, since it is possible that the positive PCR assay resulted from turtle blood contamination within the feeding leech. In addition, at least 84 mosquito species have been documented in Florida [51], some of which are known to feed on reptiles [52]. However, our evaluation of the wet versus dry season (*Season*; Field dataset), along with our frequency histogram (Fig 2), was not indicative of a disease transmission pattern strongly associated with a vector species that displays seasonal trends. Alternatively, if the virus is transmitted by a vector with year-round abundance, then we would expect a more uniform frequency histogram compared to the peaks that occur during the first quarter of the calendar year. In addition to the positive significance of the *CL7* variable, the findings described above provide further support for direct virus transmission, and we suggest that initial studies should be designed to examine this route.

As depicted in Fig 2, 70.2% (33/47) of the positive turtles were collected during the months of January through March. The peaks in positive cases are only partially explained by the timing of our intentional field surveys. Surveys were performed at the initial mortality site along the St Johns River during the months of March, April, and May 2018, after which the detection of sick ceased. Following a resurgence of reports in early 2019, surveys were performed in January and February of that year. Of the 33 positive cases detected during the months of January through March, only 13 (39.4%) were a result of intentional survey efforts. With the exception of three cases that were provided by wildlife rehabilitation centers, the remaining TFV1-positive cases collected during this timeframe came from citizen reports via one of the three hotline options described earlier. Therefore, although our surveys increased the detection of sick and dead turtles during the early months of the year, we still believe that the data portrayed in the frequency histogram is an accurate representation of the timing of the course of clinical disease associated with TFV1. As we suggest below with respect to our **Weather** dataset models, the apparent temporal patterns of clinical disease may be related to unevaluated factors associated with ambient temperatures.

Our **Necropsy** dataset analyses revealed that *AnyPlaques* and its interaction term with *Species* were significant predictors for TFV1 infection status. In softshells, plaques were often quite severe, covering approximately 50–100% of the oral or cloacal mucosa. In addition, two positive softshells (190506-01 and 200910-01) also had extensive plaques within the length of their small and large intestine segments. In contrast, the oral plaques observed in three positive hardshell turtles were less severe and presented as discrete patches that covered <15% of the mucosal surface. Based on these findings, when faced with multiple mortality investigations and restricted financial resources, molecular testing could be prioritized for hardshells, since necropsy findings are less indicative of TFV1 status in these species than in softshells.

Our **Necropsy** models indicated that *Sex*, *Maturity*, and *PL* were not significant predictors of TFV1 status. With respect to softshells, these variables are somewhat intertwined, in that mature females are often larger and therefore have longer plastron lengths than mature males. We did not include plastron length in the models with sex or maturity to avoid these confounding effects. In general, there was a collection bias towards adult (*n* = 53) as compared to subadult (*n* = 10) softshells. However, because we detected two subadult (sexually immature) softshells that were positive for TFV1, we can conclude that if sexual virus transmission occurs, this is not the sole route of transmission, and that the manifestation of clinical disease is not limited to breeding phenology.

There are several possibilities that might explain why sick and dead turtles collected during cooler temperatures were more likely to test positive. One explanation is that the TFV1 virus may have a preferred temperature range, within which it can more readily replicate and cause clinical disease in its turtle host. In red-eared sliders experimentally infected with the ranavirus FV3 (frog virus 3), environmental temperature affected turtle survival and disease progression [42]. Compared to turtles maintained at 28°C (82.4°F), those kept at 22°C (71.6°F) had lower survival rates and developed more severe clinical signs and microscopic lesions, leading the authors to suggest that the higher temperature may confer a protective effect against disease. Although such studies have not been performed for TFV1, the virus was successfully cultured in flasks maintained at 25°C (77°F) [12].

Another possibility is that the apparent TFV1-temperature association may be due to underlying factors related to turtle physiology and immunology. As ectotherms, turtles and other reptiles are unable to mount an internal fever in response to infection. To compensate, reptiles will attempt to increase their temperature via behavioral actions commonly known as “fever basking.” This may be one reason why turtles with clinical signs of TFV1 were commonly found hauled out along the shoreline of a water body [12]. Furthermore, reptile physiology (e.g., metabolism, effective immune function, and reproduction) is intricately tied to a species-specific temperature range, often referred to as the preferred optimal temperature zone (POTZ). Based on experimentally controlled temperature studies, the POTZ for many turtles falls between 25° and 33°C (77°-91.4°F) [53–56]. Of the 47 positive turtles, we found that 25 (53.2%) were collected with an associated *xTmax* of <25°C, and that 35 (74.5%) were collected at *xTmax* <27°C. Temperature is thought to strongly influence the immune system in ectothermic animals, with colder temperatures often resulting in decreased immune function [57–59]. Additionally, in many reptile species, the thymus involutes during the winter months in response to increasing levels of glucocorticoid (i.e., corticosterone) hormones [60]. In turn, thymus involution causes a temporary decrease in T cell maturation and an inability to mount an antibody response [57,60].

Our two **Spatial** models demonstrated that geographical and temporal factors were significant in predicting the probability that a turtle would test positive. *Target* turtles within a 5-km distance of *associated* positive turtles were more likely to test positive for TFV1, and the likelihood of a *target* turtle testing positive slightly increased if it was also collected within +/-30 days of *associated* positive turtles. Turtle movements vary seasonally, with both sexes moving greater distances during warmer temperatures and females moving greater distances during the nesting period [18,47]. Thus, if an individual turtle were exposed to the virus during the warmer months or during nesting forays, and it became an asymptomatic carrier, it might travel greater distances than it would during other times of the year. However, once a turtle becomes clinically ill, its movements are probably reduced, especially across terrestrial environments. Therefore, the linear distances that turtles traveled prior to collection were likely influenced by temperature, health status, and other unknown factors, such as human-assisted movement.

The initial 2018 turtle mortalities were observed in March in the Upper St. Johns River, which flows south to north. By mid-May, positive turtles were detected at Crescent Lake (connected to the Lower St. Johns River via Dunns Creek), a straight-line distance of approximately 117 km to the north. Puzzlingly, a single positive softshell (181102-01) was collected in April from Collier County, which represents a completely distinct watershed (Big Cypress HUC8 watershed). Although the means of virus transmission are still not fully understood, our **Spatial** analyses provide a general understanding of the risk of TFV1 transmission relative to previously detected positive turtles and thereby may help to inform guidance to managers for future surveillance efforts.

## Conclusion and recommendations

In this work, we evaluated TFV1 infection status and available metadata using an epidemiological framework. Although it is clear that hallmarks of TFV1 infection include histopathological brain lesions and neurological abnormalities [12], our basic understanding of this disease still remains in its infancy. Therefore, the intention of our work was to help guide future research and surveillance efforts.

The results of this study can be used to formulate Florida-specific guidelines for TFV1 continued surveillance and management actions. The response (e.g., field investigations, necropsy, and laboratory diagnostics) to reported freshwater turtle mortalities may vary based upon a given scenario and goals, especially when faced with limited resources. For example, when more than one sick or dead turtle is reported from a single location within a seven-day period, and if the mortality event occurs during the colder months (see Fig 2) in a zone where TFV1 has been previously detected, then the likelihood of TFV1 as a cause of mortality may be increased. Furthermore, if cloacal and oral plaques are observed in softshell turtles, this finding could further suggest TFV1 infection and financial resources could be conserved by pursuing limited confirmatory testing. In contrast, when investigating freshwater turtle mortality events in geographic regions where TFV1 has not yet been detected, and in turtle species with unknown virus susceptibility or less prominent gross lesions (e.g., hardshell species), greater investment of logistical and financial resources may be required. When developing management actions at the landscape level, a 5-km distance from identified TFV1 infection sites might be the minimum buffer zone. Importantly, with the detection of TFV1 virus in urine samples, as well as the intense staining of renal tissue via *in situ* hybridization [12], virus transmission via shedding in urine is possible. Therefore, when establishing buffer zones, connections among waterbodies that facilitate movement of turtles and contaminated water should also be considered. In Florida, the extensive system of constructed retention ponds and connecting canals in some urbanized areas (e.g., Brevard County) may justify classifying the entire localized system at-risk for TFV1-associated mortality events once the presence of the virus is documented.

We can offer several points for consideration regarding the impact on freshwater turtle populations and future directions of study. First, our mortality observations of large, female softshell turtles in a vitellogenic or gravid reproductive state are concerning. For common snapping turtles (*Chelydra serpentina*), another large-bodied and long-lived freshwater turtle species that can be infected with TFV1 (V. Guzman-Vargas, pers comm, 4/18/22), population stability depends upon the survival of adult, reproductive females, which do not reach sexual maturity until 12 years-of-age [61]. The unique reproductive strategy of the Florida softshell (i.e., capacity to lay multiple clutches of eggs in a breeding season, possibly as early as five-years-old) [40] may afford some protection against adverse population effects of TFV1. However, due to the lack of baseline population values for Florida softshells, it will be difficult to discern disease impacts, and, for other less fecund susceptible host species, the impacts may be more dire. Secondly, as has been suggested for a ranavirus [42], turtles may facilitate the spread of TFV1 across the landscape if they are able to chronically and asymptomatically maintain virus infection before succumbing to comorbidities or trauma. We identified two turtles for which the proximate cause-of-death was vehicular trauma, but TFV1 was also detected. The infection may have led to a higher risk of vehicular trauma, as occurs with other neurological diseases in wildlife [62]. Alternatively, these turtles may have been in the early stage of disease, or chronic/asymptomatic TFV1 carriers, and would have continued to transmit the virus to other individuals if not struck on the road. Third, our characterization of cases as clustered mortality events is compatible with a highly transmissible waterborne virus that is shed in urine or other secretions. Therefore, we suggest that future research should prioritize the study of potential direct transmission.

To conclude, our data highlight how rapidly an emerging wildlife disease can spread across the landscape. The spatial progression of TFV1 may have occurred via multiple means, such as natural animal movement, human-assisted movement, or virus-contaminated fomites. We advocate that turtle releases and translocations should be restricted to situations where disease screening can be performed, in order to avoid environmental pathogen transmission among habitats and host species. Information on the environmental stability and transfer of the TFV1 virus is currently lacking; yet, the identification and spread of TFV1 in peninsular Florida provide further validation for the strict implementation of biosecurity practices recommended to mitigate inadvertent transfer of other aquatic pathogens [63,64].

## Supporting information

S1 Text This supporting text provides additional manuscript details under three subheadings.
(1) Weather Dataset: Describes the methods used to analyze weather data obtained from the National Oceanic and Atmospheric Administration (NOAA) and the Community Collaborative Rain, Hail and Snow Network (CoCoRaHS). (2) Spatial Dataset: Describes the methods used to construct Least Cost Paths (LCP) between pairs of turtles; these LCP values were used as distance values in the spatial analyses. (3) Ancillary Analyses: Describes additional analyses used to examine relationships between post-mortem condition and carcass treatment.
(DOCX)

S1 Table Turtle dataset.
This file provides data values (Data tab) used in analyses for 117 freshwater turtles necropsied from 9 March 2018 until 5 September 2021 in Florida, USA. The Variable Key tab provides descriptions of each variable presented in the Data tab. This table is also available at the following repository link: https://f50006a.eos-intl.net/F50006A/OPAC/Details/Record.aspx?BibCode=5991926
(XLSX)

S2 Table Logistic regression model set results used to determine the best Field, Necropsy, and Weather model that describes the probability of turtles in Florida, USA, testing positive for Turtle fraservirus 1 (TFV1) via PCR. Variables included in the Field model set include turtle species (*Species*; hardshell, softshell), *Urbanicity* (low, medium, high), season (*Season*; wet, dry), and simple cluster (*CL7*; yes, no). Variables included in the Necropsy model set include *Species*, sex (*Sex*; male, female), presence of cloacal and/or oral plaques (*AnyPlaques*; yes, no), age (*Age*; adult, subadult), and plastron length (*PL*; cm). Variables included in the Weather model set include average maximum temperature (*xTmax*), average minimum temperature (*xTmin*), average precipitation (*Precip*), and day-length (*DL*; hours). Parameters in the table include AIC_c_ = Akaike’s Information Criterion adjusted for small sample size, ∆AIC_c_ = relative difference in AIC_c_ between each model and the model with the lowest AIC_c_, W_*i*_ = Akaike weight for model *i*, K = number of parameters, and -LogLik = Negative log-likelihood of the model. Model set denoted by the dataset column.
(DOCX)

S1 Fig Aggregated (within 2 km) Turtle fraservirus 1 (TFV1)-positive and -negative turtles depicted with least cost paths between all tested animals and water delineated in the National Wetlands Inventory (NWI).
(TIF)

S2 Fig Least cost path calculated between two turtles in a less urban setting that tested positive for Turtle fraservirus 1 (TFV1) via PCR. Movement is prioritized through water across choices in eight cardinal directions (within a 100-m grid).
(TIF)

S3 Fig Least cost path calculated between two turtles in a more urban setting that tested positive for Turtle fraservirus 1 (TFV1) via PCR. Movement is prioritized through water across choices in eight cardinal directions (within a 100-m grid). The discrepancy between the contemporary landscape (imagery from March 2021) and water features documented in the National Wetlands Inventory (NWI) is illustrated.
(TIF)

S1 Appendix Steps followed to download precipitation data from the Community Collaborative Rain, Hail, and Snow Network (CoCoRaHS). (DOCX)

S2 Appendix Steps followed to download NOAA station temperature and precipitation data. (DOCX)
