# Correction: Epidemiological factors associated with Turtle fraservirus 1 (TFV1) in freshwater turtles in Florida, USA

**DOI:** 10.1371/journal.pone.0332639

**Published:** 2025-09-16

**Authors:** Lisa A. Shender, Andrea Sylvia, Brian A. Stacy, Veronica Guzman-Vargas, Thais C.S. Rodrigues, Pedro Viadanna, Jordan A. Vann, Kylle Cahill-Patray, Thomas B. Waltzek, Kuttichantran Subramaniam, Brittany Bankovich

The tenth author’s name is spelled incorrectly. The correct name is: Kuttichantran Subramaniam.
