# Supplementary material for: Epidemiological factors associated with Turtle fraservirus 1 (TFV1) in freshwater turtles in Florida, USA
Source: PLoS One. 2025 Apr 1;20(4):e0320097. doi: 10.1371/journal.pone.0320097 (PMC11960915; doi:10.1371/journal.pone.0320097)
Supplement: S1 Text — (1) Weather Dataset: Describes the methods used to analyze weather data obtained from the National Oceanic and Atmospheric Administration (NOAA) and the Community Collaborative Rain, Hail and Snow Network (CoCoRaHS). (2) Spatial Dataset: Describes the methods used to construct Least Cost Paths (LCP) between pairs of turtles; these LCP values were used as distance values in the spatial analyses. (3) Ancillary Analyses: Describes additional analyses used to examine relationships between post-mortem condition and carcass treatment. (DOCX) [file pone.0320097.s001.docx]

**S1 Text**

**Climate Dataset**

The process of downloading CoCoRaHS and NOAA data is not immediately intuitive, and we have therefore provided detailed steps in S1 and S2 Appendices, respectively. CoCoRaHS is a community-based network of volunteers who receive training on how to install, monitor, and transmit data collected from program-provided high-quality manual rain gauges. With more than a 20-year history and major sponsorship from the NOAA, CoCoRaHS data is widely used by The National Weather Service, the United States Department of Agriculture, mosquito control districts, and many others. Once the data were downloaded, we pulled up the relevant 30-day date range for each turtle. We next evaluated if the quality of the data was sufficient to keep the dataset (S1 Appendix provides examples of this process). First, we removed stations if precipitation values were present in <90% of the 30-day date range, where missing data was represented by a double hyphen (--). Next, we carefully considered datasets that included multi-day values. A multi-day value was provided as a numeric value followed by an asterisk (e.g., 0.03*), which represented the accumulated sum of precipitation for that day and all preceding days noted with a double asterisk (**). For datasets that fully contained the multi-day values (i.e., the start and end date of the 30-day period had a numeric value), the dataset was considered complete, and the station was retained for analyses. However, the presence of multi-day values at the start or end date of the 30-day period required closer scrutiny. If the first date in the date range had a double asterisk, then the station was only retained if there were no double asterisk entries for the dates preceding the first date or if the corresponding multi-day value was a 0.00*. If the last date in the date range had a double asterisk, then the station was only retained if the corresponding multi-day value beyond the last date was a 0.00* value. Finally, for each retained station, we summed the precipitation values (including the multi-day values) across the 30 days. We then calculated the average total precipitation value across all included stations for each turtle to obtain the *Precip* variable used in our Climate Dataset analyses.

We created a dataframe of 50 NOAA weather stations. We included 44 stations that were located within the 24 Florida counties from which turtles were collected, as well as six additional stations in neighboring counties. We downloaded available temperature (maximum and minimum daily temperatures) and daily precipitation data from these 50 stations from January 1, 2018, to the end of September 2021. For each turtle in our dataset that had a GPS collection location, we then identified the nearest NOAA weather station based on the station GPS values. Through this process, we ended up with a list of 31 NOAA stations at distances that ranged from 0.81 miles (1.3 km) to 36.98 miles (59.5 km) from the turtles, with an average distance of approximately 11 miles (17.8 km). We averaged the daily maximum temperatures (values provided under the NOAA Tmax column header) across a 30-day period (as described in manuscript text) to determine the *xTmax* value used in our climate analyses. We converted the NOAA-provided temperature values from Fahrenheit to Celsius and the precipitation values from mm to cm for our analyses.

**Spatial Dataset**

To represent movement distances between pairs of individual turtles, we generated least cost paths, identified as the routes of least resistance between turtle collection locations. We used ArcMap 10.3.1 (ESRI, 2015) to create a study-wide raster surface representing resistance to turtle movement. Since aquatic turtles move more efficiently through water, we coded all surface water (includes many urban retention ponds) and wetland polygons in the National Wetlands Inventory (NWI; USFWS 2017) as a 1. This national database was used to ensure reproducibility of our analytical methods with future research. We acknowledge there are inconsistencies between the NWI and the current landscape at fine scales, particularly in recently developed areas (see Figures S3D-E). We coded all other land cover types as 2 (CLC version 3.3, FWC/FNAI). We then created a binary raster from the combined polygon layers (where water = 1 and land = 2), which we reduced to a 100-meter resolution to accommodate memory restrictions in processing least cost paths. This allowed the least cost path algorithm to identify water as being twice as efficient for turtle movement than land, and thus prioritize paths through water. Next, we created the eight-directional (four adjacent or four diagonal raster cells) resistance surface rasters (package ‘gdistance’, R version 4.2.2). We then calculated pairwise least cost paths between every combination of turtle locations in the dataset (package ‘gdistance’, R version 4.2.2). Figure S1 portrays the least cost paths between all possible turtle pairs. Figures S2 and S3 depict fine-scale examples of distances between paired turtles and aim to better demonstrate how the least cost paths were calculated through grid cells at different spectrums of urbanization.

**Ancillary Analyses**

The molecular detection of viral pathogens in animal tissue can be negatively influenced by factors that may degrade nucleic acids (DNA or RNA), such as freeze-thaw cycles and post-mortem condition. The freshwater turtles necropsied in our study were collected under a variety of field conditions and logistical constraints. Some turtles were necropsied soon after their euthanasia and had no tissue autolysis; however, the post-mortem condition of deceased turtles collected in the field varied, and these turtles were often frozen prior to necropsy. Therefore, we wanted to determine if post-mortem condition and freezing were associated with test status. We evaluated the probability of a positive TFV1 PCR result with a set of logistic regression models using the independent variables of carcass treatment (frozen: no = 0, yes = 1) and post-mortem condition (none/minor = 0, moderate/severe = 1). We tested additive and interactive models and used Akaike’s information criterion for small sample sizes (AIC_c_) to determine the most plausible model. We considered carcass treatment and post-mortem condition to be significant if the 95% confidence interval of the parameter estimate did not overlap zero. All species (softshells, cooters, and sliders) were combined in the analysis.

The analysis included all 93 turtles for which TFV1 RT-PCR test results were available (data shown in S1 Table). The additive model of carcass treatment and post-mortem condition was supported (ΔAIC_c_ = 0.00, weight = 0.74) over the interactive model. Turtles that were frozen prior to necropsy were less likely to test positive for TFV1, regardless of their post-mortem condition (β_0_ = 1.15 ± 0.44, β_preservation frozen_ = -1.27 ± 0.49). With post-mortem condition held constant at the none/minor autolysis or decomposition category, the predicted probability of a non-frozen turtle resulting in a positive PCR assay was 0.76 [0.57, 0.88 95% CI] compared to a probability of 0.47 [0.33, 0.62 95% CI] for a frozen turtle. There was no support for difference in the probability of a positive PCR assay with respect to post-mortem condition (β_moderate/severe decomposition_ = -0.69 ± 0.47). When carcass treatment status was held constant as not frozen, the probability of a turtle with none/minor autolysis or decomposition resulting in a positive PCR assay was 0.76 [0.57, 0.88 95% CI] and the probability for a turtle with moderate/severe decomposition was predicted at 0.61 [0.36, 0.82 95% CI]. Variation was high for the estimated effect sizes of both variables and the results may be influenced by unequal numbers of samples per group or additional confounding variables that we were unable to analyze, such as the length of time a turtle was stored in the freezer. In addition, turtles collected from sites that required a round-trip drive of more than four hours were often frozen before they could be transported to Gainesville for evaluation, and these turtles were more likely to have come from locations beyond the counties where TFV1 was initially reported.
