# Supplementary material for: Epidemiological factors associated with Turtle fraservirus 1 (TFV1) in freshwater turtles in Florida, USA
Source: PLoS One. 2025 Apr 1;20(4):e0320097. doi: 10.1371/journal.pone.0320097 (PMC11960915; doi:10.1371/journal.pone.0320097)
Supplement: S2 Table — Variables included in the Field model set include turtle species (Species; hardshell, softshell), Urbanicity (low, medium, high), season (Season; wet, dry), and simple cluster (CL7; yes, no). Variables included in the Necropsy model set include Species, sex (Sex; male, female), presence of cloacal and/or oral plaques (AnyPlaques; yes, no), age (Age; adult, subadult), and plastron length (PL; cm). Variables included in the Weather model set include average maximum temperature (xTmax), average minimum temperature (xTmin), average precipitation (Precip), and day-length (DL; hours). Parameters in the table include AICc = Akaike’s Information Criterion adjusted for small sample size, ∆AICc = relative difference in AICc between each model and the model with the lowest AICc, Wi = Akaike weight for model i, K = number of parameters, and -LogLik = Negative log-likelihood of the model. Model set denoted by the dataset column. (DOCX) [file pone.0320097.s003.docx]

**Table S2.** Logistic regression model set results used to determine the best Field, Necropsy, and Weather model describing the probability of turtles in Florida, USA, testing positive for Turtle fraservirus 1 (TFV1) by RT-PCR. Variables included in the Field model set include turtle species (*Species*; hardshell, softshell), *Urbanicity* (low, medium, high), season (*Season*; wet, dry), and simple cluster (*CL7*; yes, no). Variables included in the Necropsy model set include *Species*, sex (*Sex*; male, female), presence of cloacal and/or oral plaques (*AnyPlaques*; yes, no), age (*Age*; adult, immature), and plastron length (*PL*; cm). Variables included in the Weather model set include average maximum temperature (*xTmax*), average minimum temperature (*xTmin*), average precipitation (*Precip*), and day-length (*DL*; hours). Parameters in the table include AIC_c_ = Akaike’s Information Criterion adjusted for small samples size, ∆AIC_c_ = relative difference between the particular model and the best model, Wi = Akaike weight, K = number of parameters, -LogLik = Negative loglikelihood of the model. Model set denoted by the dataset column.

| **Dataset** | **Model** | **Predictor Variables** | **K** | **-LogLik** | **AIC_c_** | **ΔAIC_c_** | **W_i_** |
| --- | --- | --- | --- | --- | --- | --- | --- |
| Field | 1 | *CL7* | 2 | -43.34 | 90.83 | 0.00 | 0.20 |
|  | 2 | *CL7 + Species* | 3 | -42.45 | 91.17 | 0.35 | 0.17 |
|  | 3 | *CL7 + Urbanicity* | 4 | -41.37 | 91.21 | 0.39 | 0.16 |
|  | 4 | *CL7 + Species + Species X CL7* | 4 | -41.76 | 92.00 | 1.17 | 0.11 |
|  | 5 | *CL7 + Season* | 3 | -42.97 | 92.23 | 1.40 | 0.10 |
|  | 6 | *CL7 + Season + Urbanicity* | 5 | -40.80 | 92.33 | 1.50 | 0.09 |
|  | 7 | *CL7 + Season + Species* | 4 | -41.98 | 92.43 | 1.60 | 0.09 |
|  | 8 | *CL7 + Species + Urbanicity* | 5 | -40.96 | 92.64 | 1.81 | 0.08 |
|  | 9 | *Species + Season + Urbanicity* | 5 | -52.48 | 115.69 | 24.86 | 0.00 |
|  | 10 | *Species + Urbanicity* | 4 | -53.89 | 116.25 | 25.43 | 0.00 |
|  | 11 | *Season + Urbanicity* | 4 | -54.29 | 117.06 | 26.23 | 0.00 |
|  | 12 | *Urbanicity* | 3 | -55.66 | 117.61 | 26.79 | 0.00 |
|  | 13 | *Season + Species* | 3 | -58.38 | 123.04 | 32.22 | 0.00 |
|  | 14 | *Species* | 2 | -59.58 | 123.30 | 32.47 | 0.00 |
|  | 15 | *Season* | 2 | -60.57 | 125.27 | 34.45 | 0.00 |
|  |  |  |  |  |  |  |  |
| Necropsy | 1 | *AnyPlaques + Species + AnyPlaques X Species* | 4 | -38.44 | 85.42 | 0.00 | 1.00 |
|  | 2 | *PL + Species + PL X Species* | 4 | -50.13 | 108.78 | 23.37 | 0.00 |
|  | 3 | *Age + Species* | 3 | -51.79 | 109.90 | 24.48 | 0.00 |
|  | 4 | *Age* | 2 | -53.02 | 110.20 | 24.78 | 0.00 |
|  | 5 | *Age + Species + Sex* | 4 | -51.76 | 112.05 | 26.63 | 0.00 |
|  | 6 | *Age + Sex* | 3 | -53.02 | 112.34 | 26.93 | 0.00 |
|  | 7 | *Species* | 2 | -55.36 | 114.88 | 29.47 | 0.00 |
|  | 8 | *Sex* | 2 | -55.83 | 115.81 | 30.397 | 0.00 |
|  | 9 | *Sex + Species* | 3 | -55.33 | 116.98 | 31.56 | 0.00 |
|  |  |  |  |  |  |  |  |
| Weather | 1 | *xTmax* | 2 | -57.28 | 118.70 | 0.00 | 0.37 |
|  | 2 | *xTmin* | 2 | -57.94 | 120.02 | 1.32 | 0.19 |
|  | 3 | *xTmax + DL* | 3 | -57.04 | 120.36 | 1.66 | 0.16 |
|  | 4 | *xTmax + Precip* | 3 | -57.28 | 120.84 | 2.14 | 0.13 |
|  | 5 | *xTmin + Precip* | 3 | -57.92 | 122.12 | 3.42 | 0.07 |
|  | 6 | *xTmin + DL* | 3 | -57.93 | 122.15 | 3.44 | 0.07 |
|  | 7 | *DL* | 2 | -60.44 | 125.01 | 6.31 | 0.02 |
|  | 8 | *Precip* | 2 | -61.24 | 126.62 | 7.92 | 0.01 |
