# Supplementary figures and images for: Epidemiological factors associated with Turtle fraservirus 1 (TFV1) in freshwater turtles in Florida, USA
Source: PLoS One. 2025 Apr 1;20(4):e0320097. doi: 10.1371/journal.pone.0320097 (PMC11960915; doi:10.1371/journal.pone.0320097)

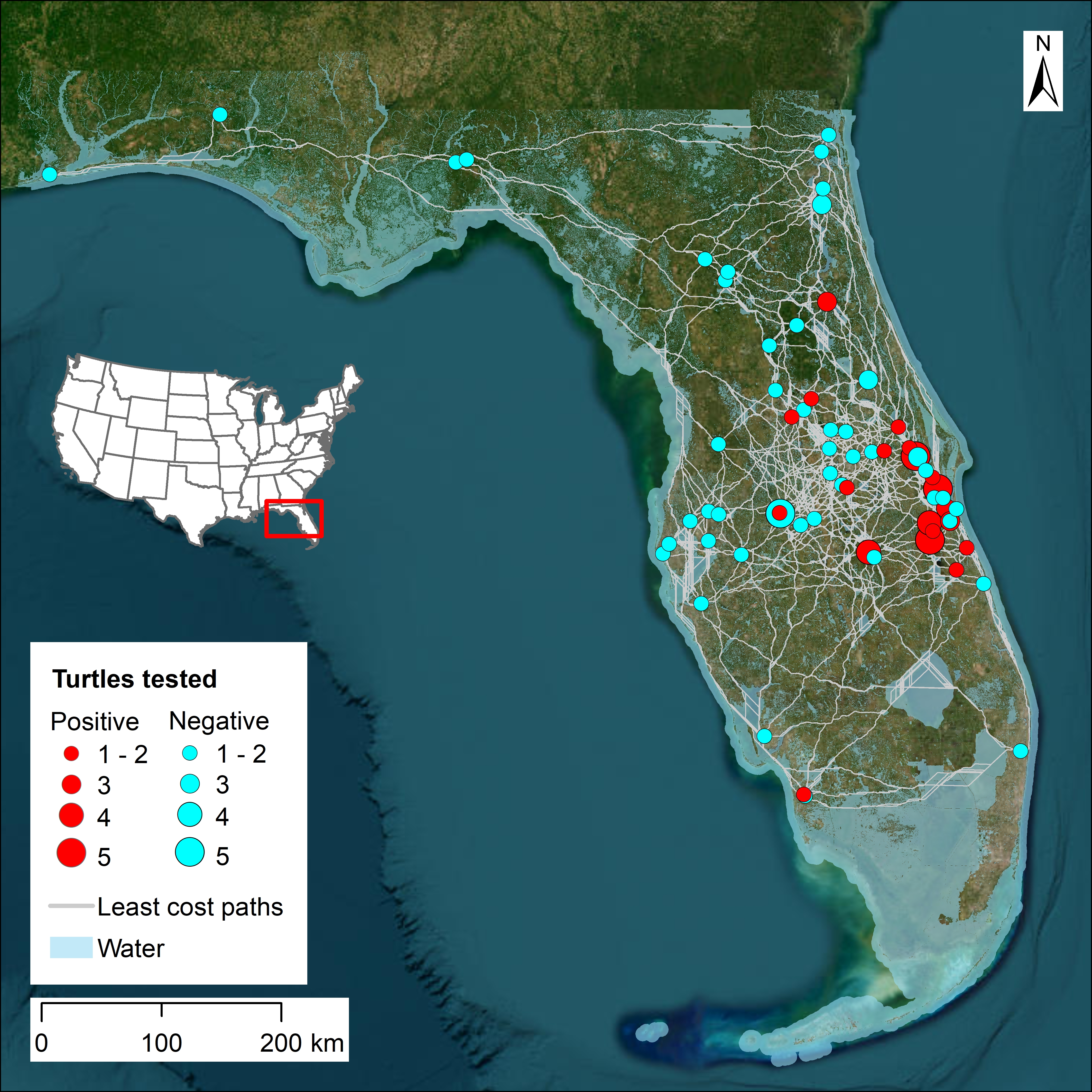

Supplement: S1 Fig — Aggregated (within 2 km) Turtle fraservirus 1 (TFV1)-positive and -negative turtles depicted with least cost paths between all tested animals and water delineated in the National Wetlands Inventory (NWI). (TIF) [file pone.0320097.s004.tif]

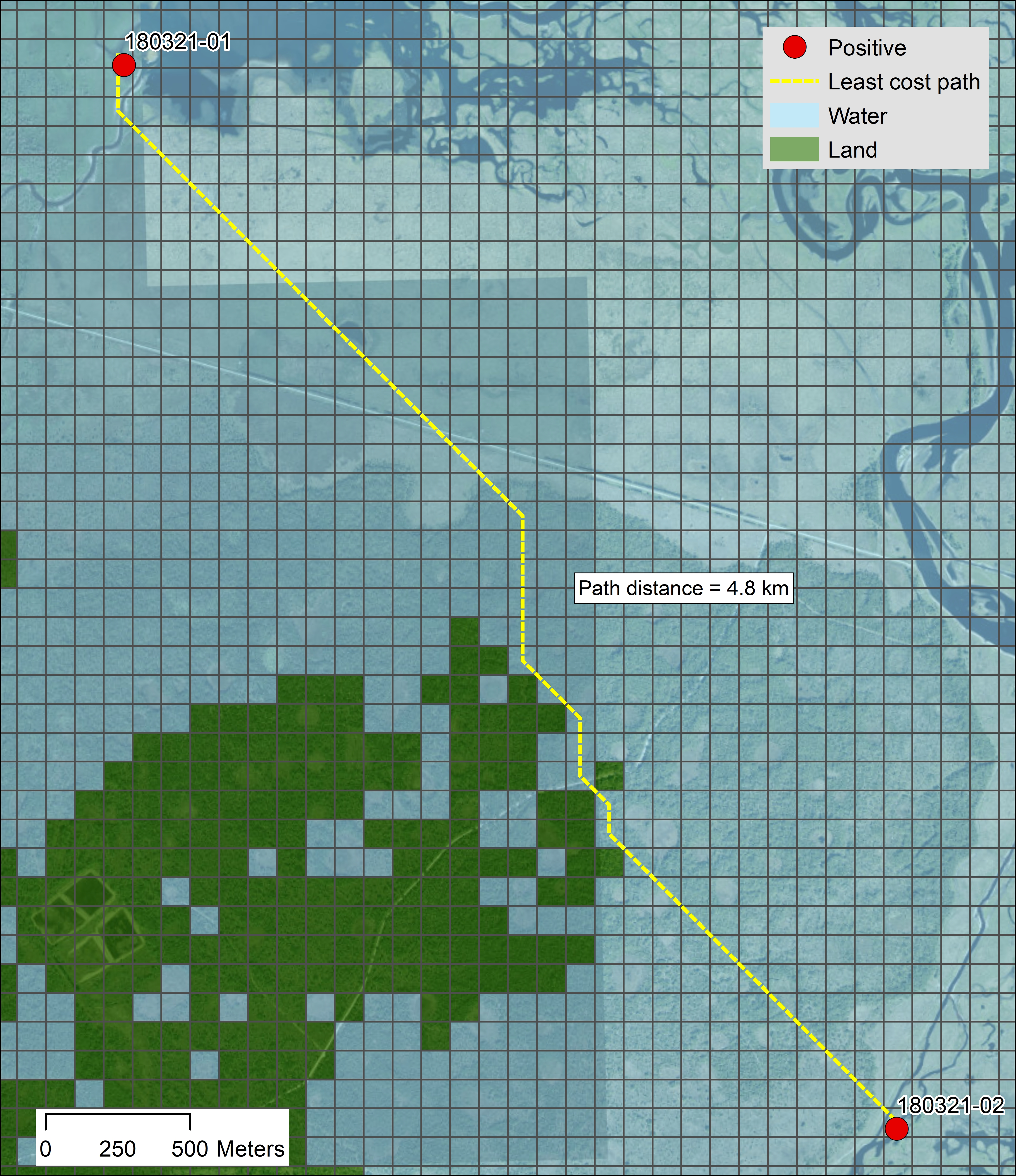

Supplement: S2 Fig — Least cost path calculated between two turtles in a less urban setting that tested positive for Turtle fraservirus 1 (TFV1) via PCR. Movement is prioritized through water across choices in eight cardinal directions (within a 100-m grid). (TIF) [file pone.0320097.s005.tif]

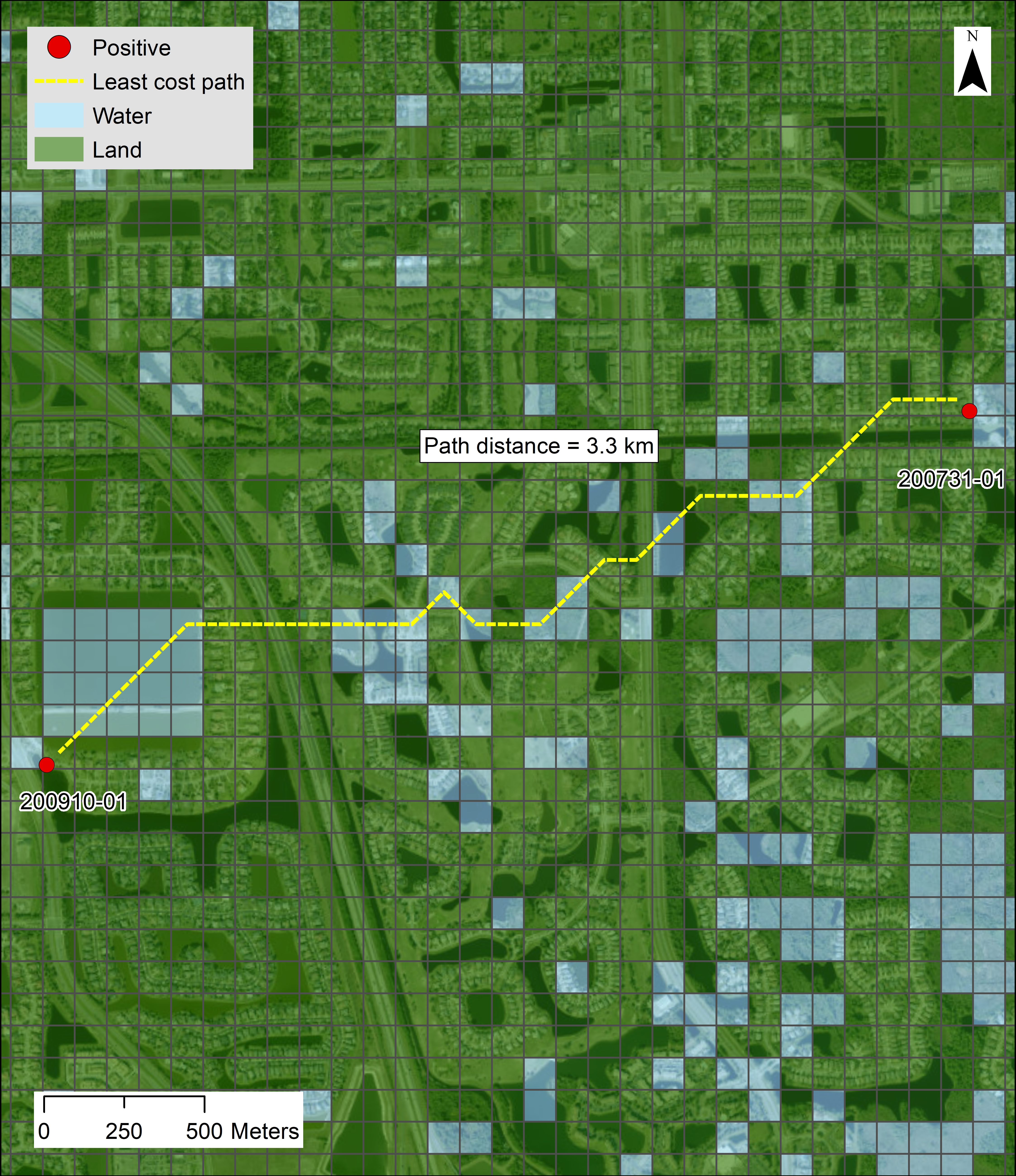

Supplement: S3 Fig — Least cost path calculated between two turtles in a more urban setting that tested positive for Turtle fraservirus 1 (TFV1) via PCR. Movement is prioritized through water across choices in eight cardinal directions (within a 100-m grid). The discrepancy between the contemporary landscape (imagery from March 2021) and water features documented in the National Wetlands Inventory (NWI) is illustrated. (TIF) [file pone.0320097.s006.tif]
