# Supplementary material for: Epidemiological factors associated with Turtle fraservirus 1 (TFV1) in freshwater turtles in Florida, USA
Source: PLoS One. 2025 Apr 1;20(4):e0320097. doi: 10.1371/journal.pone.0320097 (PMC11960915; doi:10.1371/journal.pone.0320097)
Supplement: S1 Appendix — (DOCX) [file pone.0320097.s007.docx]

**S1 Appendix: Steps followed to download CoCoRaHS station data for this research**

1. Opened Community Collaborative Rain, Hail and Snow Network website: <https://www.cocorahs.org/>
2. Used List Stations link found under View Data column on left: <https://www.cocorahs.org/Stations/ListStations.aspx>
3. Selected Florida from drop down menu of States.
4. Performed iterative process of relevant County selection from drop down menu to generate complete list of CoCoRaHS stations for each county of interest.
5. Created data file for each county, whereby each station’s GPS location was included in file data. The latitude and longitude data for each station was manually pulled from the View Station Detail option for each station.
6. Imported county data files into R program and ran code to identify all stations within a 10-mile (~16.1 km) radius of each turtle’s GPS location in the dataset.
7. For all identified stations, used the Station Precipitation Summary link (<https://www.cocorahs.org/ViewData/StationPrecipSummary.aspx>) to enter the station name and retrieve data for the date range of 01 January 2018 to 31 December 2021.
8. Saved data by copying the generated table into an Excel file (there was no mechanism to download a .csv file from the Station Precipitation Summary link).
9. Pulled station precipitation data for each turtle’s associated 30-day date range and evaluated if the data quality was sufficient to use in analyses, as described in S1 Text.
