# Supplementary material for: Epidemiological factors associated with Turtle fraservirus 1 (TFV1) in freshwater turtles in Florida, USA
Source: PLoS One. 2025 Apr 1;20(4):e0320097. doi: 10.1371/journal.pone.0320097 (PMC11960915; doi:10.1371/journal.pone.0320097)
Supplement: S2 Appendix — (DOCX) [file pone.0320097.s008.docx]

**S2 Appendix: Steps followed to download NOAA station data for this research**

1. Opened National Centers for Information NOAA website: <https://www.ncei.noaa.gov/maps/daily-summaries/>
2. Used the tool bar in Layers tab on left to select the date of 2018/03/01. This brought up all stations with data as far back as the selected date.
3. Clicked Update Map button at bottom of Layers tab.
4. In the map pane on right, used the zoom button to zoom in on the state of Florida. Yellow dots indicated NOAA stations.
5. Selected the *Wrench* icon in the upper right of the Layers tab. This brought up a Daily Observations Tools pop-up window.
6. Selected the *Identify* button in the pop-up window above. This enabled the mouse to select NOAA station yellow dots.
7. Clicked on a yellow dot. This opened up a specific NOAA station on the left side in the Results tab.
8. Selected the View Station Details below the NOAA station name. This opened up a new web browser tab with station details including: Name, NetworkID, Latitude/Longitude, and period of coverage. This page also informed what data types were available at each station, such as Air Temperature and Precipitation.
9. From here, selected the *Add to Cart* button. Once added, in the upper right corner of the web browser window, there appeared a “**Cart (Free Data)**” box.
10. Selected the cart and then selected the data Output Format as daily CSV option.
11. Used the calendar options to select the date range for the data to download.
12. Selected the Continue button.
13. Option to select Standard or Metric units.
14. Selected data types of Precipitation and Air Temperature.
15. Selected the Continue button.
16. Entered an email address to receive data file.
17. Selected the Submit Order button.
18. Reviewed new screen that appeared to confirm request was successfully submitted.
19. Receive confirmation email immediately.
20. Within minutes to hours, received another email that said “Order Complete” and contained a link to download the CSV file of data. Downloaded file promptly, before link expired.
